# Comparison of Sutureless Aortic Valve Replacement and Transcatheter Aortic Valve Implantation: A Systematic Review and Meta-Analysis of Propensity Score Matching

**DOI:** 10.31083/j.rcm2511391

**Published:** 2024-11-04

**Authors:** Shidong Liu, Hao Chen, Wenjun Zhou, Pengying Zhao, Liang Qi, Yalan Zhang, Bing Song, Cuntao Yu

**Affiliations:** ^1^The First Clinical Medical College of Lanzhou University, 730000 Lanzhou, Gansu, China; ^2^Department of Cardiovascular Surgery, First Hospital of Lanzhou University, 730013 Lanzhou, Gansu, China; ^3^Department of Cardiovascular Surgery, Fuwai Hospital, National Center for Cardiovascular Diseases, Chinese Academy of Medical Sciences, Peking Union Medical College, 100006 Beijing, China

**Keywords:** transcatheter aortic valve implantation, sutureless aortic valve replacement, meta-analysis, propensity score matching, mortality

## Abstract

**Background:**

To evaluate the clinical outcomes of sutureless aortic valve replacement (SUAVR) and transcatheter aortic valve implantation (TAVI).

**Methods:**

We systematically searched the electronic database and the Clinical Trials Registry up to 31 February 2023. Random effects model risk ratio (*RR*) and mean differences (MD) with corresponding 95% confidence intervals (CIs) were pooled for the clinical outcomes.

**Results:**

The included 16 studies using propensity-matched analysis consisted of 6516 patients, including 3258 patients in the SUAVR group and 3258 patients in the TAVI group. The SUAVR group had lower mortality than the TAVI group at 1-year [*RR* = 0.53, 95% CI (0.32, 0.87), *I*^2^ = 49%, *p* = 0.01], 2-year [*RR* = 0.56, 95% CI (0.37, 0.82), *I*^2^ = 51%, *p* = 0.03] and 5-year [*RR* = 0.56, 95% CI (0.46, 0.70), *I*^2^ = 0%, *p* < 0.01]. The SUAVR group had a significantly lower rate of new permanent pacemaker implantation (PPI) [*RR* = 0.74, 95% CI (0.55, 0.99), *I*^2^ = 48%, *p* = 0.04], moderate-to-severe paravalvular leak (PVL) [*RR* = 0.18, 95% CI (0.11, 0.30), *I*^2^ = 0%, *p* < 0.01], more-than-mild residual aortic regurgitation (AR) [*RR* = 0.27, 95% CI (0.14, 0.54), *I*^2^ = 0%, *p* < 0.01]. In addition, the SUAVR group had a higher rate of new-onset atrial fibrillation (AF) [*RR* = 3.66, 95% CI (1.95, 6.89), *I*^2^ = 84%, *p* < 0.01], major or life-threatening bleeding event [*RR* = 3.63, 95% CI (1.81, 7.28), *I*^2^ = 83%, *p* < 0.01], and higher postoperative mean aortic gradient [*MD* = 1.91, 95% CI (0.73, 3.10), *I*^2^ = 91%, *p* < 0.01] than the TAVI group.

**Conclusions:**

The early and mid-term clinical outcomes of SUAVR were superior compared to TAVI. Further studies should be conducted to highlight the specific subgroups of patients. that will benefit from each technique.

**INPLASY Registration Number:**

INPLASY 2022110058 (https://inplasy.com/inplasy-2022-11-0058/).

## 1. Introduction

In the treatment of patients with aortic valve stenosis (AS), the prognoses of surgical aortic valve replacement (SAVR) are reproducible and well established [1]. However, transcatheter aortic valve implantation (TAVI) has been introduced into surgical practice as new alternative treatment in the last ten years, which has shown favorable clinical and hemodynamic results in AS patients at intermediate or high surgical risk [2, 3], and which is expanding to low-risk patients [4, 5].

The lack of removal of the diseased and calcified aortic valve tissue, resulting in an increased risk of post-operative complications, has been acknowledged as a major limitation of TAVI [6, 7, 8]. With the development of stentless valves, sutureless aortic valve replacement (SUAVR) has been proposed as another treatment option for AS patients to overcome these limitations of TAVI [9]. Sutureless valves are made of biological tissue and can be quickly implanted, which reduces aortic cross-clamp and cardiopulmonary bypass time compared to conventional sutured valves [10, 11], and facilitates the use of a newer less invasive surgical techniques [12]. Thus, SUAVR is expected to reduce postoperative complications and improve the quality of life for AS patients.

SUAVR may potentially have a patient cohort similar to TAVI, therefore the objective of this systematic review and meta-analysis was to evaluate the clinical outcomes of SUAVR and TAVI.

## 2. Methods

### 2.1 Date Source and Search Strategy

We systematically searched electronic databases (PubMed, Cochrane library, EMbase and MEDLINE) and the Clinical Trials Registry (https://www.clinicaltrials.gov) until February 31, 2023. The search strategy used the combination of “Surgical Procedure, Sutureless”, “Sutureless technique”, “Sutureless Surgical Procedures” and “Transcatheter Aortic Valve Implantation”, “Transcatheter Aortic Valve Replacement”, “TAVI”, “TAVR” with no restrictions on language. References from the reviewed studies were also screened to identify additional articles.

### 2.2 Eligibility Criteria

Studies were included if they met the following criteria, based on patient, intervention, comparison, endpoint, and study design: (1) the patient underwent SUAVR or TAVI with no restrictions on surgical risk, (2) the intervention was SUAVR regardless of the valve type, (3) the comparison group was TAVI regardless of the valve style, (4) primary endpoint: mortality at various follow-up periods. Secondary outcomes: moderate-to-severe paravalvular leak (PVL), more-than-mild residual aortic regurgitation (AR), myocardial infarction (MI), major vascular complication, new permanent pacemaker implantation (PPI), stroke, new renal replacement therapy, new-onset atrial fibrillation (AF), major or life-threatening bleeding event, postoperative mean aortic gradient, intensive care unit (ICU) length of stay, cross-clamp time and cardiopulmonary bypass (CPB) time. Primary endpoints were defined based on the Valve Academic Research Consortium-2 definitions and major or life-threatening bleeding event were defined based on the Bleeding Academic Research Consortium (BARC), (5) comparative studies with propensity-matched analysis.

### 2.3 Study Selection and Data Extraction

Two authors (PZ and WZ) independently screened studies using the following criteria: (1) removal of duplicates; (2) selection of titles and abstracts based on inclusion/exclusion criteria; (3) evaluation of eligibility by reading the full text; (4) determination of included studies. Disagreements were resolved by discussions with a third reviewer (SL) or by consensus. This study was a systematic review and meta-analysis, and therefore ethical and patient approval was not required.

The study data were extracted by two authors (LQ and YZ) independently and included: first author, publication year, study design, number of patients, patient demographics, medical history, procedural characteristics, outcomes and follow-up time. Data were then revised by a third reviewer (HC) for accuracy. Discrepancies regarding data incorporation were resolved by consensus among all authors.

### 2.4 Quality Assessment and Statistical Analysis

The quality of the included studies were evaluated by the Newcastle-Ottawa Scale (NOS) [13] including: (1) patient selection, (2) comparability of the study groups, and (3) the assessment of outcomes.

Categorical variables were denoted by numbers and percentages, and continuous variables were reported as standardized mean and standard deviation. The pooled results of dichotomous endpoints were estimated by risk ratio (*RR*) and 95% confidence intervals (CIs) with the Mantel-Haenszel method. The pooled data of continuous endpoints were assessed by mean differences (MD) with 95% CIs with the inverse-variance method.

Heterogeneity assessments were performed using χ^2^-based *Q* statistics and *I*^2^ tests. A *p* < 0.10 or *I*^2^ > 50% were considered as significant heterogeneity. The *RR*s and *MD*s were combined using the random effect model. As a sensitivity analysis, by excluding one study in each turn, we analyzed the results in the presence of heterogeneity to assess the robustness and potential effect modifiers. The publication bias was evaluated visually using funnel plots, and Peter’s or Egger’s test [14, 15] was used to quantify publication bias. *p* < 0.05 was considered statistically significance in this meta-analysis. All pooled analyses were calculated using the R version 4.1.1 (R Foundation for Statistical Computing, Vienna, Austria).

## 3. Results

### 3.1 Study Characteristics and Quality Assessment

**Fig. 1.**
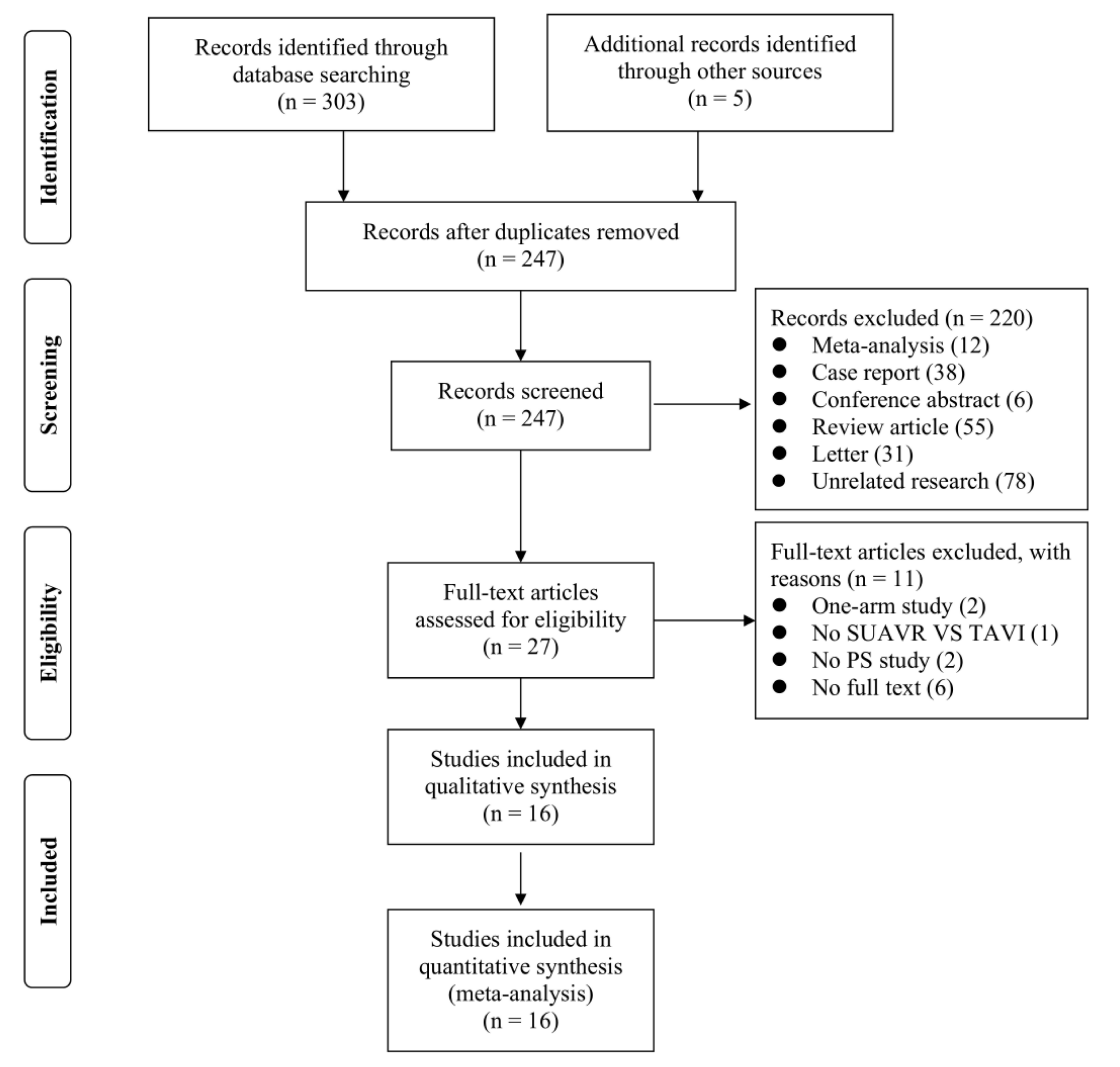
**Literature Selection Flowchart**. SUAVR, sutureless aortic valve replacement; TAVI, transcatheter aortic valve implantation; PS, propensity score matching.

308 studies were included in the preliminary search, of which 27 studies were potentially relevant and the full text was read. Finally, a total of 16 studies were included [16, 17, 18, 19, 20, 21, 22, 23, 24, 25, 26, 27, 28, 29, 30, 31] (Fig. 1). After propensity-matched analysis, 3258 SUAVR and 3258 TAVI patients were included in each group for a total of 6516 patients. The patient demographics and quality assessment of the included studies are reported in Table 1 (Ref. [16, 17, 18, 19, 20, 21, 22, 23, 24, 25, 26, 27, 28, 29, 30, 31]). Table 2 (Ref. [16, 17, 18, 19, 20, 21, 22, 23, 24, 25, 26, 27, 28, 29, 30, 31]) represents the complications of the included patients. There were no statistical differences in surgical risk between two groups including logistic European System for Cardiac Operative Risk Evaluation II (logistic Euro SCORE II) [*MD* = –0.03, 95% CI (–0.17, 0.11), *I*^2^ = 40%, *p* = 0.66] and the Society of Thoracic Surgeons score (STS score) [*MD* = –0.16, 95% CI (–0.49, 0.17), *I*^2^ = 99%, *p* = 0.33], but the SUAVR group had lower surgical risk in the Logistic EuroSCORE [*MD* = –1.12, 95% CI (–1.85, –0.40), *I*^2^ = 91%, *p* = 0.03].

**Table 1.** **Patient demographics of included studies**.

Author	Year	Number of patient		Age (years)		Male (%)		BMI (Kg/m^2^)		STS (%)		Logistic EuroSCORE		EuroScore II (%)		Newcastle-Ottawa scale
		SUAVR	TAVI	SUAVR	TAVI	SUAVR	TAVI	SUAVR	TAVI	SUAVR	TAVI	SUAVR	TAVI	SUAVR	TAVI	
D’Onofrio A *et al*. [16]	2012	38	38	80.9 ± 3.9	80.9 ± 6.9	15.8	21.1	NR	NR	NR	NR	13.7 ± 7.20	14.8 ± 7.50	NR	NR	7
Santarpino G *et al*. [17]	2014	37	37	81.5 ± 5.1	84.5 ± 5.1	40.5	48.6	NR	NR	NR	NR	18.1 ± 1.90	20.6 ± 2.20	NR	NR	7
Kamperidis V *et al*. [18]	2015	40	40	79.0 ± 4.5	79.0 ± 5.9	100	100	NR	NR	NR	NR	15.9 ± 10.60	15.5 ± 8.40	NR	NR	8
Muneretto C *et al*. [19]	2015	204	204	79.0 ± 4.0	80.0 ± 2.0	48.6	55.4	27.1 ± 2.8	26.9 ± 5.3	7.9 ± 3.20	8.2 ± 4.20	18.9 ± 5.90	19.5 ± 6.70	NR	NR	8
Santarpino G *et al*. [20]	2015	102	102	80.0 ± 4.0	79.0 ± 7.0	41.0	43.0	NR	NR	NR	NR	17.0 ± 14.0	18.0 ± 11.00	NR	NR	7
Biancari F *et al*. [21]	2016	144	144	79.4 ± 5.4	79.0 ± 6.0	38.9	37.5	NR	NR	NR	NR	NR	NR	4.1 ± 3.20	3.6 ± 2.60	8
D’Onofrio A *et al*. [22]	2016	214	214	77.4 ± 5.4	77.7 ± 7.9	35.5	35.0	27.5 ± 4.7	27.6 ± 5.2	NR	NR	10.5 ± 6.20	12.4 ± 9.10	NR	NR	8
Miceli A *et al*. [23]	2016	37	37	79.0 ± 4.5	78.8 ± 7.4	30.1	40.5	NR	NR	NR	NR	16.1 ± 11.00	15.7 ± 8.50	NR	NR	8
Bruno P *et al*. [24]	2017	30	30	79.9 ± 3.6	81.1 ± 3.3	50.0	56.7	25.8 ± 2.7	26.4 ± 2.4	NR	NR	NR	NR	5.0 ± 0.87	5.2 ± 1.15	7
Abdel-Wahab M *et al*. [25]	2020	1605	1605	75.0 ± 2.0	78.0 ± 2.0	41.7	39.7	27.8 ± 1.6	27.4 ± 1.6	2.2 ± 0.35	2.7 ± 0.45	6.20 ± 1.18	7.50 ± 1.35	NR	NR	9
Al-Maisary S *et al*. [26]	2021	52	52	75.0 ± 4.0	77.0 ± 4.3	38.0	38.0	28.0 ± 5.0	27.3 ± 5.0	3.9 ± 2.59	4.5 ± 2.76	17.0 ± 10.00	19.0 ± 12.00	NR	NR	7
Chung YH *et al*. [27]	2021	62	62	75.5 ± 5.3	76.8 ± 6.0	38.7	32.3	24.9 ± 3.3	24.9 ± 3.4	NR	NR	NR	NR	NR	NR	8
Gerfer S *et al*. [28]	2021	59	59	77.0 ± 8.0	79.0 ± 5.0	36.0	36.0	28.0 ± 6.0	28.0 ± 6.0	NR	NR	NR	NR	2.5 ± 1.20	2.5 ± 1.20	7
Vilalta V *et al*. [29]	2021	171	171	78.0 ± 5.7	77.4 ± 8.4	37.4	36.3	29.3 ± 5.0	29.2 ± 7.2	2.8 ± 0.35	2.6 ± 0.38	NR	NR	1.9 ± 0.30	1.9 ± 0.30	8
Muneretto C *et al*. [30]	2022	291	291	80.0 ± 5.0	81.0 ± 6.0	41.6	41.6	26.6 ± 1.6	26.2 ± 1.7	6.0 ± 0.93	6.0 ± 0.98	13.8 ± 1.38	13.9 ± 1.50	NR	NR	9
Santarpino G *et al*. [31]	2022	172	172	80.9 ± 5.1	79.1 ± 7.4	39.0	43.1	26.3 ± 2.9	26.7 ± 3.4	NR	NR	17.0 ± 14.0	18.0 ± 11.00	5.6 ± 2.90	6.1 ± 1.50	8

BMI, body mass index; STS, Society of Thoracic Surgeons predicted risk of mortality; EuroScore II, European System for Cardiac Operative Risk Evaluation II; NR, no reported; SUAVR, sutureless aortic valve replacement; TAVI, transcatheter aortic valve implantation.

**Table 2.** **Complication of patient population in included studies**.

Author	Year	Diabetes mellitus (%)		Hypertension (%)		Myocardial infarction (%)		Coronary artery disease (%)		Previous ICD/PPM (%)		Previous PCI (%)		Peripheral artery disease (%)		Stroke (%)		Chronic lung disease (%)		Atrial fibrillation (%)	
		SUAVR	TAVI	SUAVR	TAVI	SUAVR	TAVI	SUAVR	TAVI	SUAVR	TAVI	SUAVR	TAVI	SUAVR	TAVI	SUAVR	TAVI	SUAVR	TAVI	SUAVR	TAVI
D’Onofrio A *et al*. [16]	2012	21.0	26.0	74.0	81.0	NR	NR	34.0	45.0	NR	NR	NR	NR	13.2	18.4	1.4	1.2	13.2	21.1	15.8	13.1
Santarpino G *et al*. [17]	2014	NR	NR	73.0	59.5	27.0	37.8	NR	NR	NR	NR	NR	NR	13.5	10.8	NR	NR	18.9	32.4	NR	NR
Kamperidis V *et al*. [18]	2015	NR	NR	NR	NR	NR	NR	NR	NR	NR	NR	NR	NR	NR	NR	NR	NR	24.0	27.4	NR	NR
Muneretto C *et al*. [19]	2015	28.0	30.3	67.6	63.2	6.0	7.5	20.6	25.9	NR	NR	12.0	13.2	19.6	21	12.2	13.6	22.0	23.0	NR	NR
Santarpino G *et al*. [20]	2015	39.0	36.0	86.0	92.0	NR	NR	NR	NR	6.5	6.5	NR	NR	26.0	16.0	NR	NR	NR	NR	NR	NR
Biancari F *et al*. [21]	2016	4.2	3.5	NR	NR	3.5	2.1	NR	NR	4.9	4.9	NR	NR	8.3	9.0	NR	NR	26.4	24.3	NR	NR
D’Onofrio A *et al*. [22]	2016	27.6	27.1	88.8	74.8	NR	NR	5.6	5.1	NR	NR	NR	NR	21.5	22.4	NR	NR	18.2	16.8	NR	NR
Miceli A *et al*. [23]	2016	27.0	18.9	86.5	83.8	NR	NR	NR	NR	NR	NR	NR	NR	29.7	24.3	NR	NR	21.6	29.7	NR	NR
Bruno P *et al*. [24]	2017	20	31	83.3	73.3	10.0	6.9	NR	NR	3.3	13.8	NR	NR	40.0	30.0	NR	NR	26.7	16.7	NR	NR
Abdel-Wahab M *et al*. [25]	2020	9.5	11.2	87.6	86.8	4.9	5.0	24.5	22.4	3.7	5.9	8.6	7.5	4.2	4.2	NR	NR	6.5	8.0	10.5	9.5
Al-Maisary S *et al*. [26]	2021	42.0	40.0	87.0	87.0	9.6	15.0	NR	NR	NR	NR	17.0	44.0	15.0	29.0	NR	NR	29.0	35.0	NR	NR
Chung YH *et al*. [27]	2021	38.7	37.1	83.9	87.1	NR	NR	43.6	50.0	NR	NR	NR	NR	6.5	11.3	24.2	22.6	12.9	12.9	12.9	8.1
Gerfer S *et al*. [28]	2021	31.0	27.0	93.0	86.0	19.0	10.0	58.0	61.0	1.0	3.0	NR	NR	17.0	19.0	NR	NR	7.0	22.0	36.0	27.0
Vilalta V *et al*. [29]	2021	30.4	34.5	84.2	84.2	7.6	8.2	27.5	19.9	6.4	7.6	NR	NR	8.2	7.0	4.1	5.3	16.4	12.9	24.0	25.2
Muneretto C *et al*. [30]	2022	20.9	20.7	77.0	78.0	6.9	5.5	36.1	38.5	NR	NR	15.1	17.5	18.2	17.5	11.7	8.9	21.6	23.7	33.3	32.3
Santarpino G *et al*. [31]	2022	13.9	18.6	57.5	66.2	NR	NR	14.5	24.4	NR	NR	NR	NR	12.7	18.0	NR	NR	43.6	40.1	16.2	21.5

NR, no reported; SUAVR, sutureless aortic valve replacement; TAVI, transcatheter aortic valve implantation; ICD, implantable cardioverter defibrillator; PPM, permanent pacemaker; PCI, percutaneous coronary intervention.

### 3.2 Mortality

**Fig. 2.**
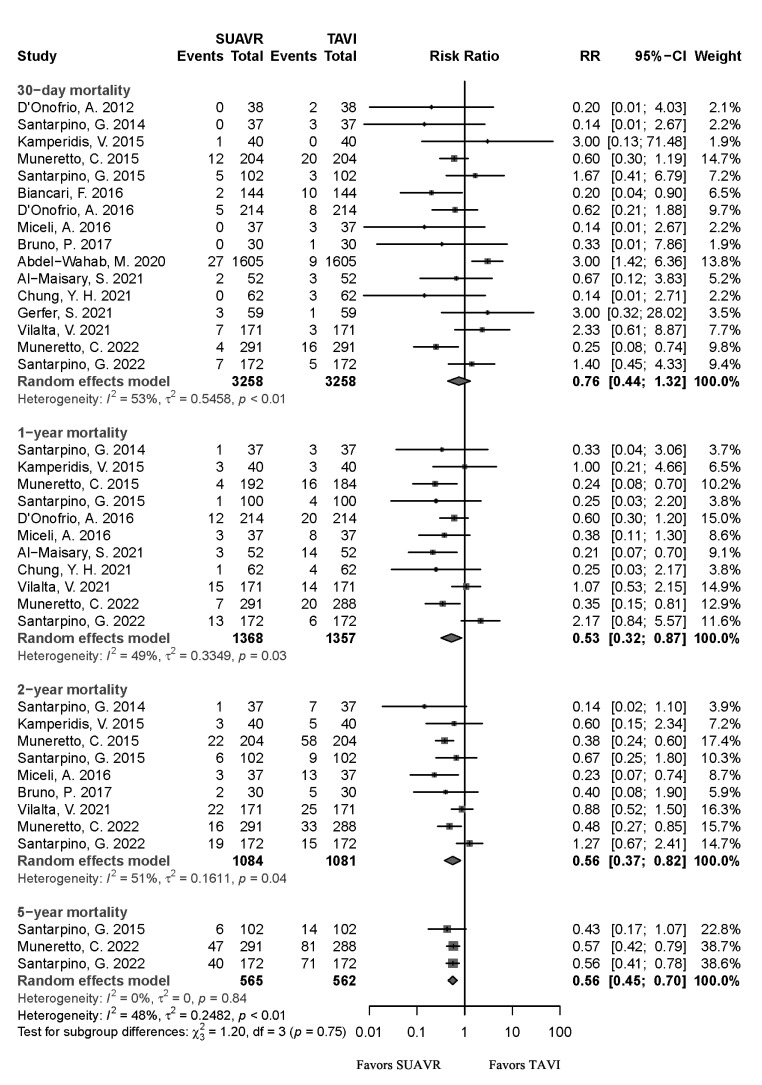
**The forest plot shows 30-day, 1-year, 2-year, and 5-year mortality risks in the SUAVR and TAVI groups, respectively**. SUAVR, sutureless aortic valve replacement; TAVI, transcatheter aortic valve implantation; CI, confidence interval; RR, risk ratio.

Pooled analysis of 16 studies showed no statistical difference in the risk for 30-day mortality [*RR* = 0.76, 95% CI (0.44, 1.32), *I*^2^ = 53%, *p* = 0.33]. However, the SUAVR group had lower mortality than the TAVI group at 1-year [*RR* = 0.53, 95% CI (0.32, 0.87), *I*^2^ = 49%, *p* = 0.01], 2-years [*RR* = 0.56, 95% CI (0.37, 0.82), *I*^2^ = 51%, *p* = 0.03] and 5-years [*RR* = 0.56, 95% CI (0.46, 0.70), *I*^2^ = 0%, *p* < 0.01] (Fig. 2).

### 3.3 Secondary Endpoints

**Fig. 3.**
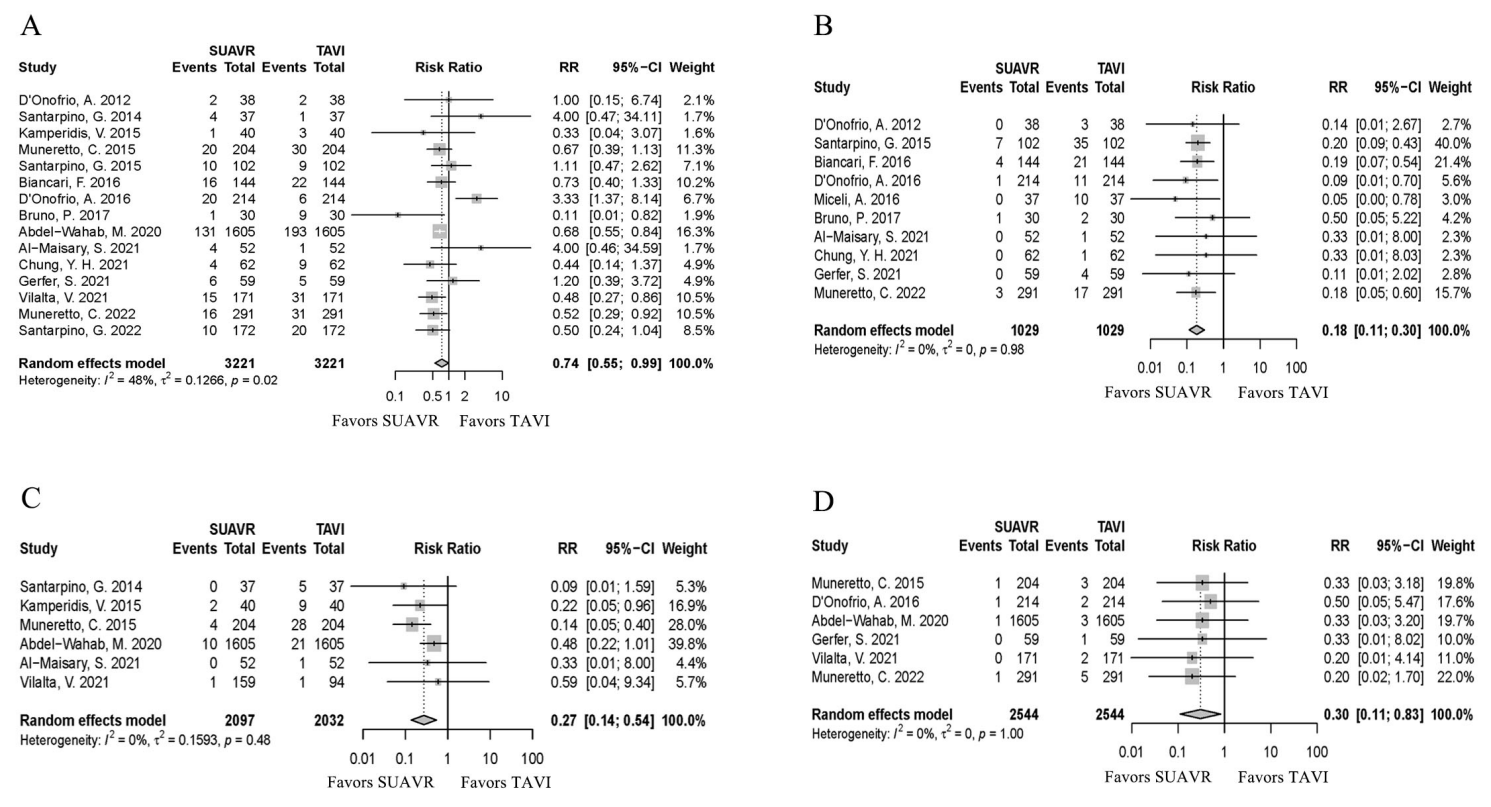
**The forest plot shows new PPI (A), moderate-to-severe PVL (B), ultra-mild residual AR (C), and MI (D) risks in the SUAVR and TAVI groups, respectively**. PPI, permanent pacemaker implantation; PVL, paravalvular leak; AR, aortic regurgitation; MI, myocardial infarction; SUAVR, sutureless aortic valve replacement; TAVI, transcatheter aortic valve implantation; RR, risk ratio; CI, confidence interval.

The SUAVR group was associated with a significantly lower rate of new PPI [*RR* = 0.74, 95% CI (0.55, 0.99), *I*^2^ = 48%, *p* = 0.04] (Fig. 3A); moderate-to-severe PVL [*RR* = 0.18, 95% CI (0.11, 0.30), *I*^2^ = 0%, *p* < 0.01] (Fig. 3B); more-than-mild residual AR [*RR* = 0.27, 95% CI (0.14, 0.54), *I*^2^ = 0%, *p* < 0.01] (Fig. 3C); MI [*RR* = 0.30, 95% CI (0.11, 0.83), *I*^2^ = 0%, *p *= 0.02] (Fig. 3D); and major vascular complications [*RR* = 0.12, 95% CI (0.07, 0.83), *I*^2^ = 0%, *p *= 0.02] than the TAVI group.

**Fig. 4.**
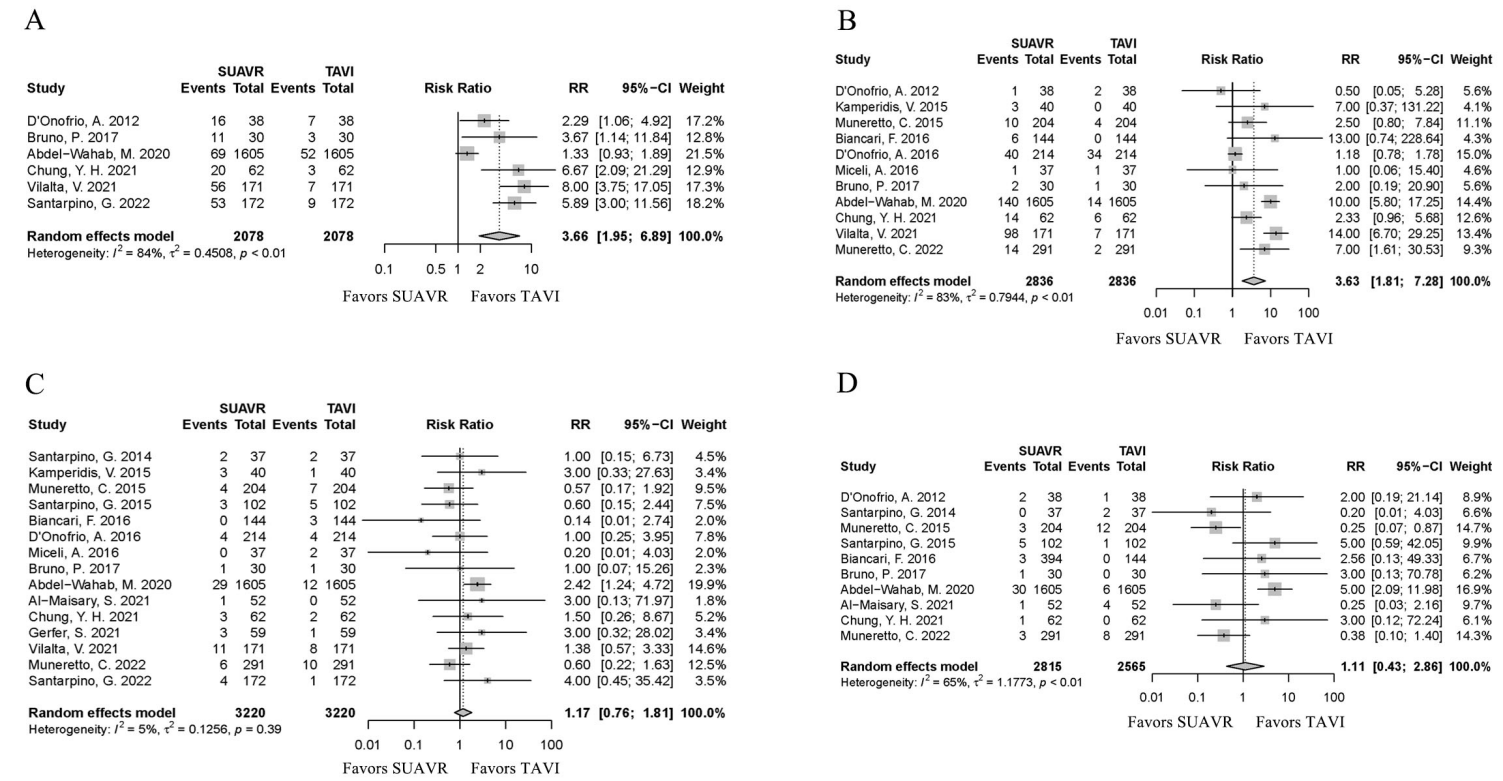
**The forest plot shows new-onset AF (A), major or life-threatening bleeding event (B), stroke (C) and new renal replacement therapy (D) risks in the SUAVR and TAVI groups, respectively**. AF, atrial fibrillation; SUAVR, sutureless aortic valve replacement; TAVI, transcatheter aortic valve implantation; RR, risk ratio; CI, confidence interval.

However, the SUAVR group had higher rate of new-onset AF [*RR* = 3.66, 95% CI (1.95, 6.89), *I*^2^ = 84%, *p* < 0.01] (Fig. 4A); major or life-threatening bleeding events [*RR* = 3.63, 95% CI (1.81, 7.28), *I*^2^ = 83%, *p* < 0.01] than the TAVI group (Fig. 4B). Additionally, there were no differences in stroke [*RR* = 1.17, 95% CI (0.76, 1.81), *I*^2^ = 5%, *p *= 0.47] (Fig. 4C) and new renal replacement therapy [*RR* = 1.11, 95% CI (0.43, 2.86), *I*^2^ = 65%, *p *= 0.83] (Fig. 4D) between two groups.

**Fig. 5.**
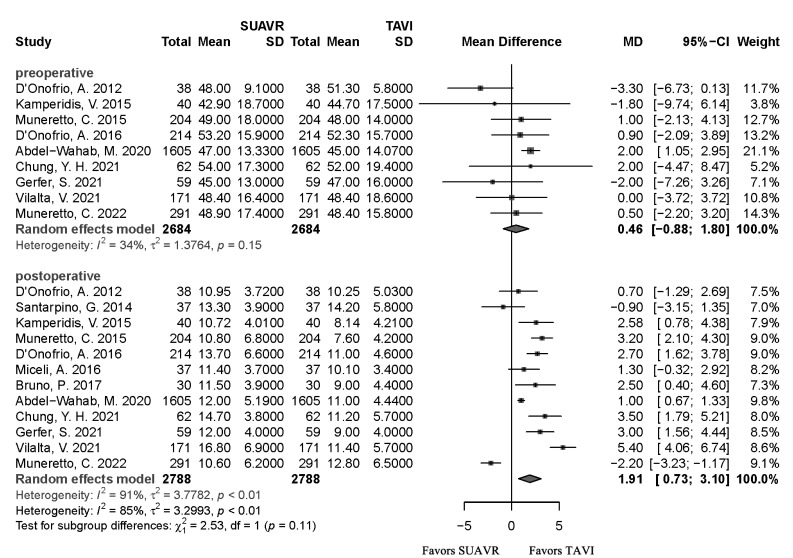
**The forest plot shows preoperative mean aortic gradient and postoperative mean aortic gradient risks in the SUAVR and TAVI groups, respectively**. SUAVR, sutureless aortic valve replacement; TAVI, transcatheter aortic valve implantation; CI, confidence interval; MD, mean difference.

**Fig. 6.**
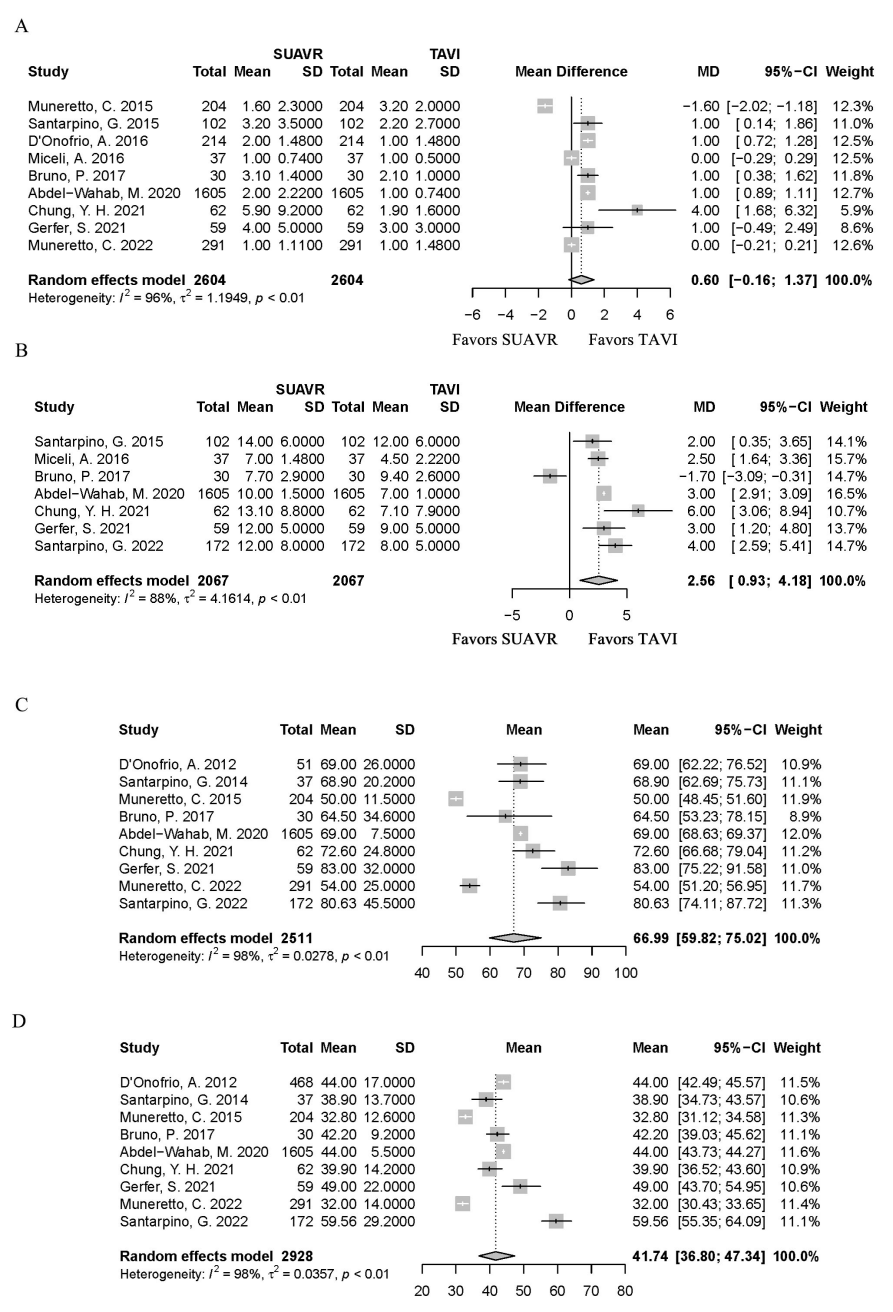
**The forest plot shows the pooled result of ICU length of stay (A), a longer length of stay (B), mean CPB time (C), and mean cross-clamp time (D), in the SUAVR and TAVI groups, respectively**. ICU, intensive care unit; CPB, cardiopulmonary bypass; SUAVR, sutureless aortic valve replacement; TAVI, transcatheter aortic valve implantation; CI, confidence interval; MD, mean difference.

There were no differences in the preoperative mean aortic gradient [*MD* = 0.46, 95% CI (–0.88, 1.80), *I*^2^ = 34%, *p *= 0.50]. However, the SUAVR group was associated with a higher postoperative mean aortic gradient [*MD *= 1.91, 95% CI (0.73, 3.10), *I*^2^ = 91%, *p* < 0.01] than the TAVI group (Fig. 5). There was no difference in the ICU length of stay [*MD *= 0.60, 95% CI (–0.16, 1.37), *I*^2^ = 96%, *p* = 0.12] (Fig. 6A), but the SUAVR group had a longer length of hospital stay [*MD *= 2.56, 95% CI (0.93, 4.18), *I*^2^ = 88%, *p* < 0.01] (Fig. 6B).

The pooled result of mean CPB time and mean cross-clamp time were 66.99 mins [95% CI (59.82, 75.02), *I*^2^ = 98%] (Fig. 6C) and 41.74 mins [95% CI (36.80, 47.34), *I*^2^ = 98%] (Fig. 6D) respectively; using the Single-arm Meta-analysis. The sensitivity analyses did not find heterogeneity in any of the studied outcomes by excluding one study in each turn (**Supplementary 1**).

### 3.4 Publication Bias

We did not find significant asymmetries in all the results obtained by the funnel plot analysis (**Supplementary 2**). The Peter’s or Egger’s test showed no publication bias in 30-day mortality (*p* = 0.12), stroke (*p* = 0.57), new PPI (*p* = 0.85), moderate-to-severe PVL (*p* = 0.50), new renal replacement therapy (*p* = 0.34), major or life-threatening bleeding events (*p* = 0.23), and postoperative mean aortic gradient (Egger’s test *p* = 0.32).

## 4. Discussion

In this systematic review and meta-analysis including 16 comparative cohort studies using a propensity-matched analysis of SUAVR and TAVI, we found that SUAVR is associated with lower mortality rates at 1, 2 and 5 years. Additionally, we observed that the SUAVR group was associated with lower rates of moderate-to-severe PVL; more-than-mild residual AR; new PPI; MI and major vascular complications. However, the SUAVR group was associated with higher rates of new-onset AF and major or life-threatening bleeding events, higher postoperative mean aortic gradients, and longer length of hospital stay.

Although there are no randomized controlled trials (RCTs) comparing TAVI and SUAVR, several RCTs have demonstrated that the majority of in-hospital complications, including all-cause mortality, stroke, and new onset renal replacement therapy, are lower in the TAVI group, compared to the conventional SAVR [3, 4, 5, 32, 33]. However, our study found lower mortality in the SUAVR group than in the TAVI group, and no differences in stroke and new renal replacement therapy, which appear to be consistent with prior meta-analyses reporting 30-day mortality [34, 35]. First, SUAVR offers faster implantation with significantly shorter CPB and cross-clamp times compared with traditional SAVR. Second, transapical procedures may have a negative effect on mortality and complications in the TAVI group. Third, the minimally invasive approach may have increased the clinical benefit of SUAVR. Finally, the potentially higher mortality in the TAVI group may be explained by a relatively older age and higher surgical risk, although these differences are not statistically significant after a propensity-matched analysis.

In the present study, the SUAVR group was associated with a lower rate of new PPI, which was similar to previous studies and meta-analyses [34, 35, 36]. Regarding the incidence of new PPI, despite the possible effects of TAVI valve selection, different TAVI approaches and implantation depth, native aortic valve removal may weaken the mechanical pressure on the His bundle region in the SUAVR group. In addition, our study also demonstrated a higher incidence of PVL and AR in TAVI patients. Prior clinical trials have reported that moderate-to-severe PVL significantly affects clinical endpoints and appears to be an independent risk factor for all-cause mortality, and that the effect of AR on mortality tends to increase proportionally with the severity of PVL [37]. The higher incidence in the TAVI group may be explained by incomplete expansion of the oversized valve, persistent calcification of the irregular aortic annulus, and calcified AV tissues [38]. However, the development of a new generation of TAVI devices that can improve the sealing between the aortic annulus has considerably reduced the incidence of significant PVL after TAVI [39, 40]. Despite accurate preoperative evaluation and controlled oversizing, TAVI had a higher incidence of PVL and residual AR when compared to SUAVR.

We also found a significantly higher rate of new-onset AF in the SUAVR group, a finding that is also consistent with previous studies [41]. There are a number of factors that may cause new-onset atrial fibrillation after cardiac surgery, such as atrial stretch, inflammation, elevated circulating catecholamines, and increased sympathetic and parasympathetic excitability [42]. The TAVI group showed a lower rate of major or life-threatening bleeding events and a shorter length of stay compared to SUAVR due to the minimally invasive approach and the absence of extracorporeal circulation. In addition, even though SUAVR can be performed via minimally invasive approaches, SUAVR is a true open-heart procedure and requires CPB and aortic cross-clamping, which can severely affect bleeding events and length of hospitalization after cardiac surgery.

Interestingly, the hemodynamic data show significantly a lower postoperative mean aortic gradient in the TAVI group compared to the SUAVR group, which may be affected by the different technical characteristics between the two groups. TAVI valves are typically selected to be oversized compared to the aortic annulus dimension in order to ensure optimal anchorage of the valve and to avoid PVL. In addition, postoperative anemia, hemodilution and inflammation may also increase the postoperative mean aortic gradient of the SUAVR group. In our single-arm meta-analysis, the pooled results for mean CPB time and mean cross-clamp time are similar to previous studies [43], but heterogeneity of the pooled results is particularly significant. These results may be related to differences between cardiac centers and clinicians, and the learning curve required for SUAVR procedures.

It should be noted that the study includes the early TAVI population, whose surgical risk may be higher, and EuroSCORE and STS scores cannot evaluate all the characteristics of decision-making, but at present, such scores can still greatly reduce the baseline difference between the two groups and obtain more credible results. It is believed that with the support of a more comprehensive evaluation mechanism and more high-quality original studies, higher quality evidence can be further obtained. Recent RCTs [3, 4, 5, 32, 33] comparing conventional SAVR with sutured valves to TAVI have reported the short-term outcomes of severe symptomatic AS patient in all surgical risk categories. While valid evidence suggests conventional SAVR and SUAVR have similar short-term efficacy, SUAVR is beneficial for minimally invasive surgery and facilitates reduced CPB and cross-clamp times [44]. A study on the potential advantages of SUAVR in high- and intermediate-risk patients, suggesting that the current patients undergoing SUAVR may be similar to the TAVI population [45].

## 5. Limitations

First, despite the absence of significant bias in baseline characteristics and the use of a propensity-matched analysis, we still cannot consider these studies to be equivalent to randomized controlled trials. Thus, the lack of RCTs is the main limitation of our meta-analysis. Second, due to the limitation of included studies, the impact of different types of TAVI and SUAVR valves, different approaches of TAVI (transfemoral or transapical) and SUAVR (mini-sternotomy, mini-thoracotomy or full sternotomy), and patients in different surgical risk categories could not be evaluated in detail. Third, patients with specific valve anatomy, such as a bicuspid aortic valve, and those undergoing concomitant other procedures, such as percutaneous coronary intervention (PCI) or coronary artery bypass grafting (CABG), were not further assessed in the present article. Fourth, STS, Logistic EuroSCORE and EuroScore II were used to evaluate the risk profile of patients in the included studies respectively, subgroup analyses based on different risk stratifications were not available, and clinical outcomes for SUAVR and TVAR in different risk categories could not be assessed. Fifth, due to the restriction of the included articles, echo data and prothesis mis-matching were not the endpoints in our meta-analysis, and Starless surgical and Evolute transcatheter aortic valve replacement (TAVR) prostheses were not compared. Sixth, the study includes the early TAVI population, whose surgical risk may be higher. Finally, although we reported ICU length of stay and hospital length of stay, additional data on cost were not available to us. Further RCTs should be performed to highlight the impact of different valve types and approaches, as well as specific subgroups of patients who may benefit from this treatment regimen.

## 6. Conclusions

The SUAVR group has better early and mid-term outcomes in mortality, PPI, PVL, AR and MI. The early and mid-term clinical outcomes of SUAVR are acceptable compared to TAVI. Further studies should be performed to highlight the specific subgroups of patients.

## Availability of Data and Materials

The datasets used and analyzed during the current study are available from the corresponding author on reasonable request.

## Supplementary Material

Supplementary material associated with this article can be found, in the online version, at https://doi.org/10.31083/j.rcm2511391.
