# Supplementary material for: Comparison of Sutureless Aortic Valve Replacement and Transcatheter Aortic Valve Implantation: A Systematic Review and Meta-Analysis of Propensity Score Matching
Source: Rev Cardiovasc Med. 2024 Nov 4;25(11):391. doi: 10.31083/j.rcm2511391 (PMC11607518; doi:10.31083/j.rcm2511391)
Supplement: Supplementary file 1 [file 2153-8174-25-11-391-s1.zip › Supplementary 1-sensitivity analyses.docx]

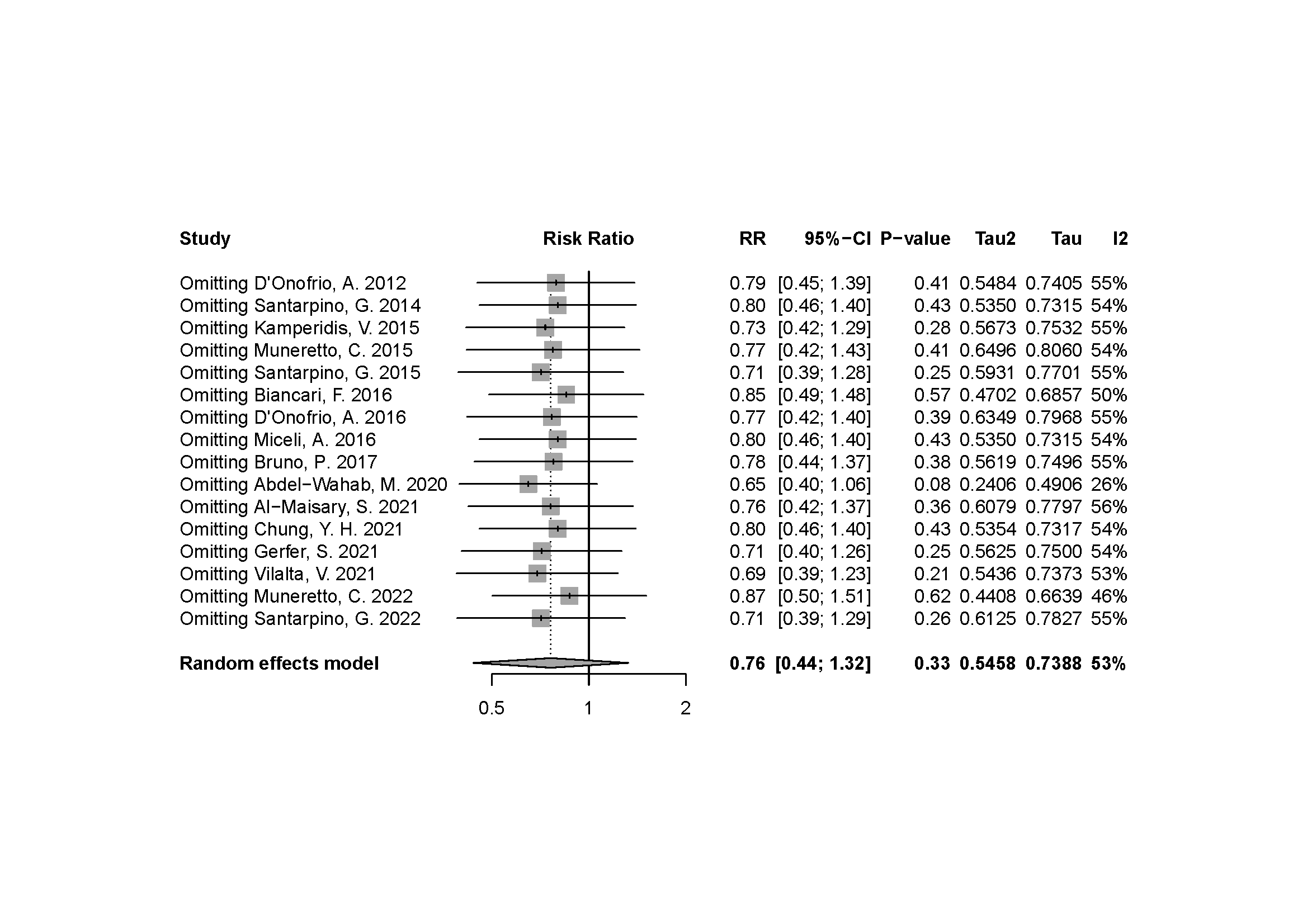


Supplementary Fig. 1 Sensitivity analyses for 30-day mortality


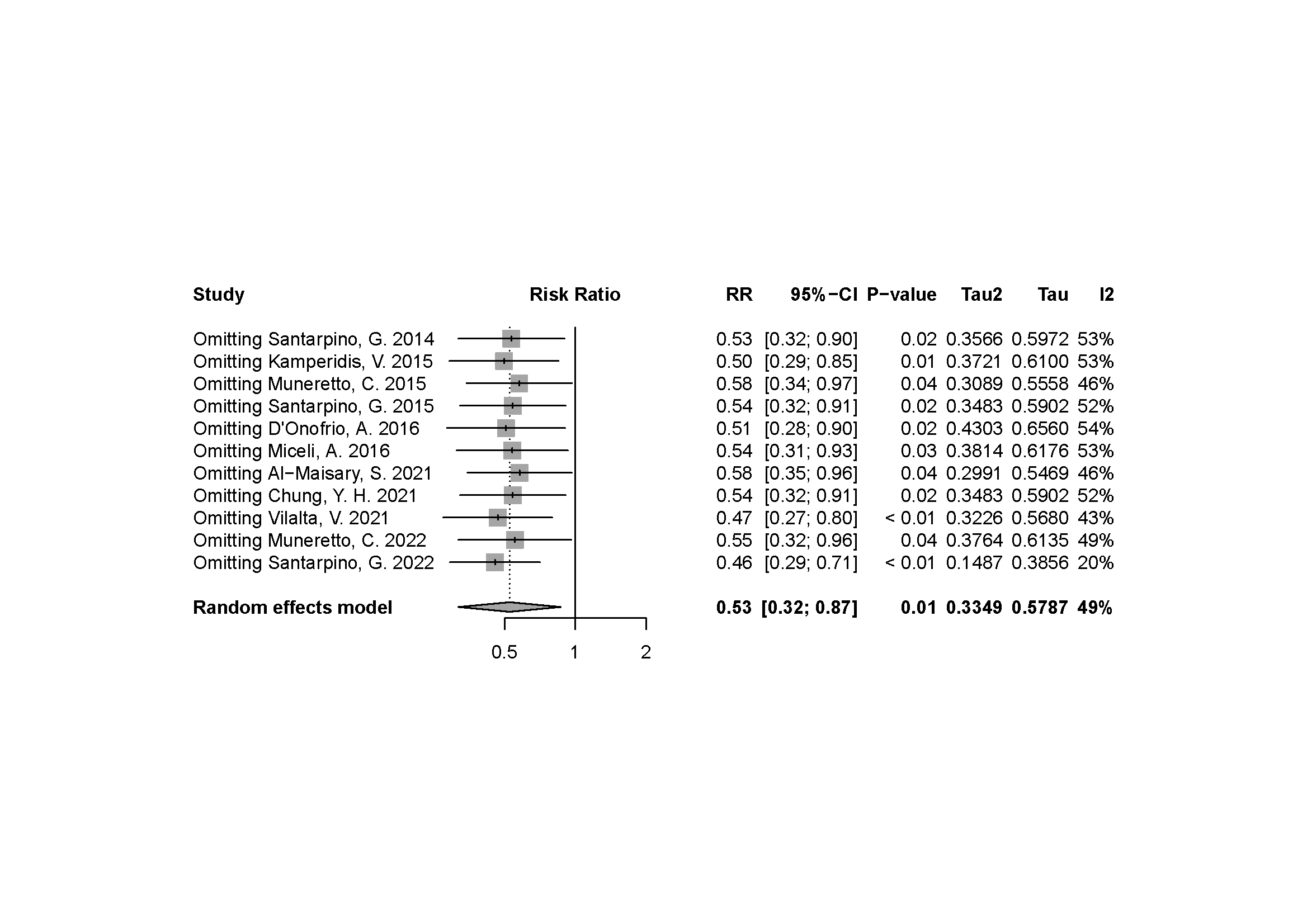


Supplementary Fig. 2 Sensitivity analyses for 1-year mortality


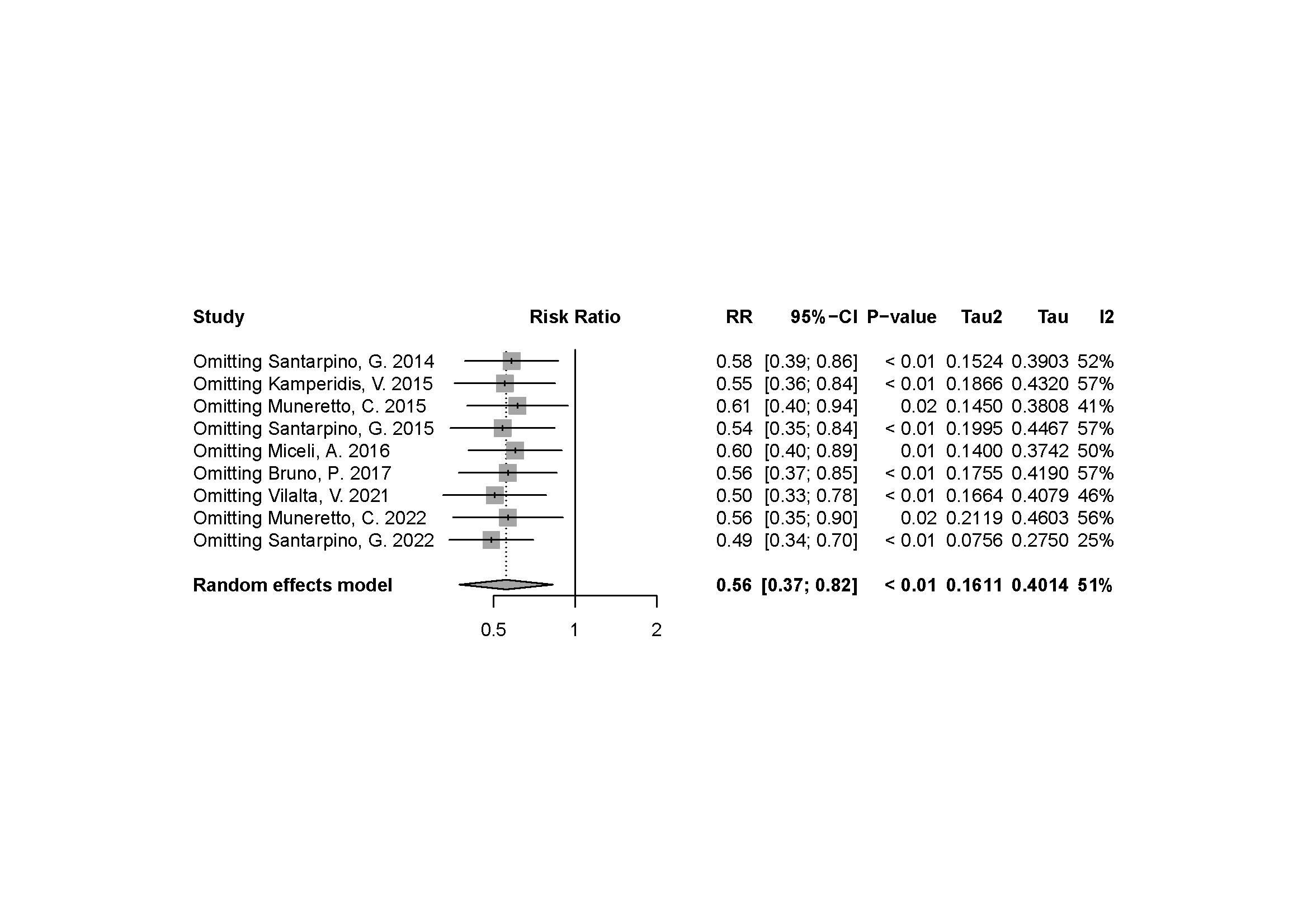


Supplementary Fig. 3 Sensitivity analyses for 2-year mortality


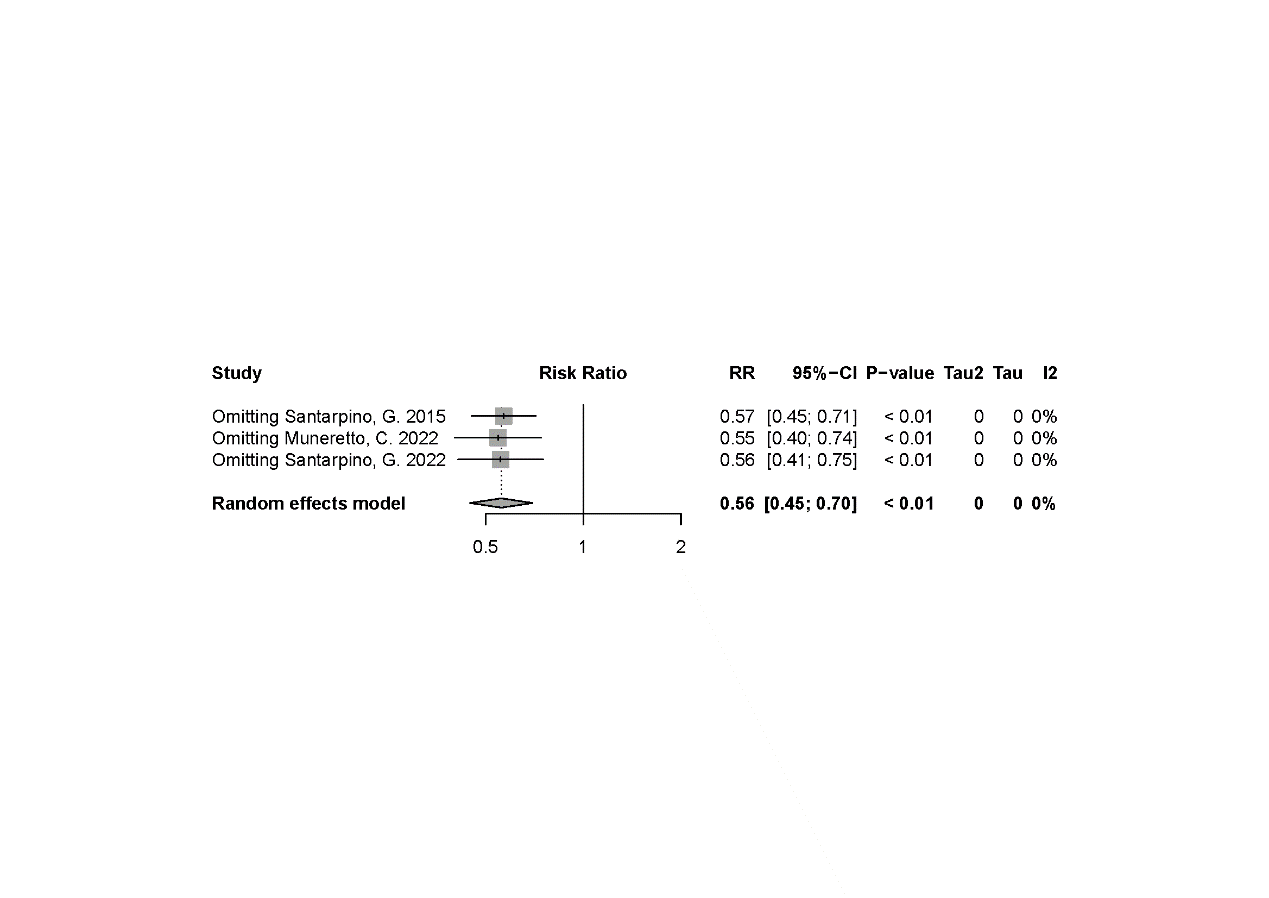


Supplementary Fig. 4 Sensitivity analyses for 5-year mortality


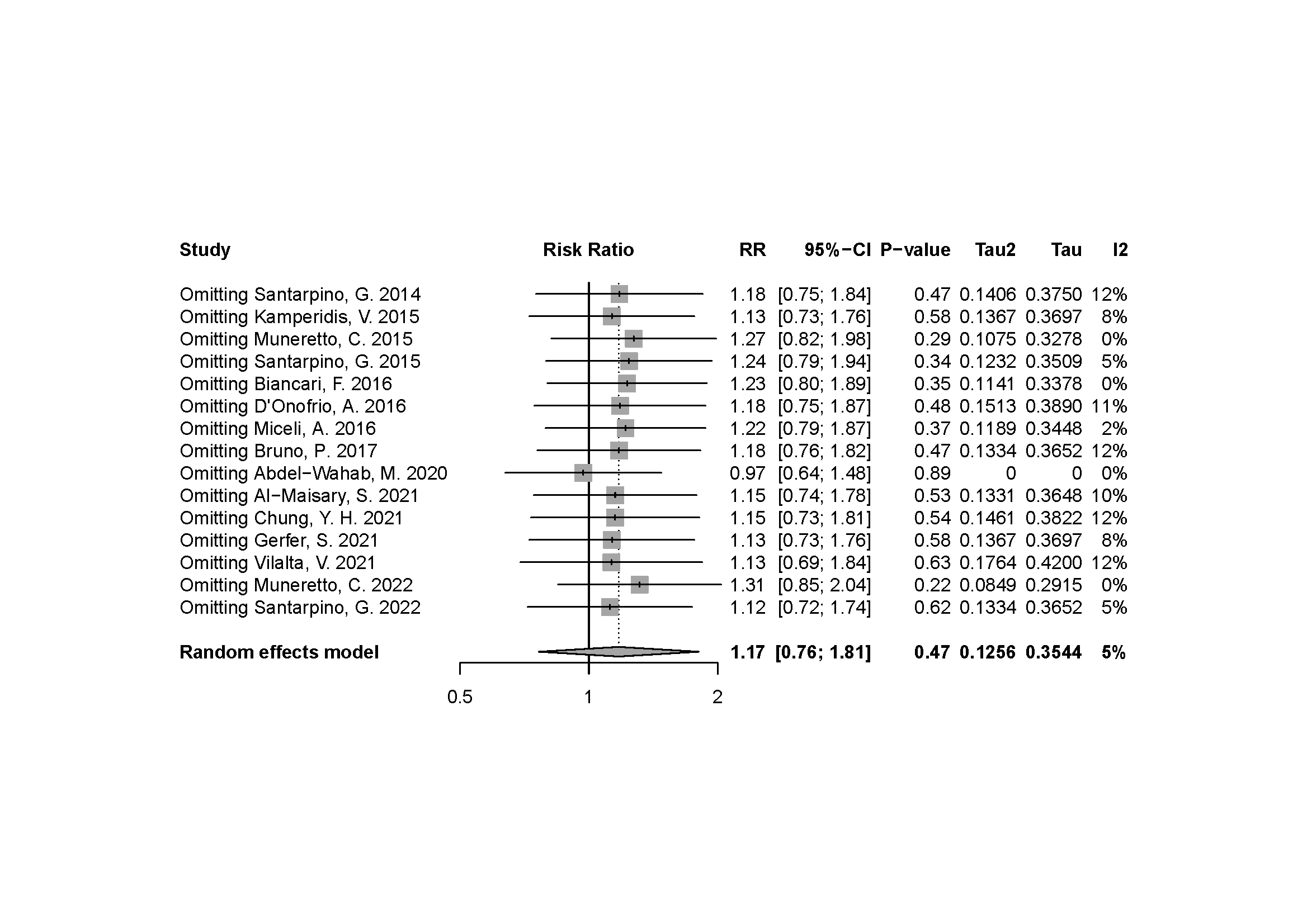


Supplementary Fig. 5 Sensitivity analyses for stroke


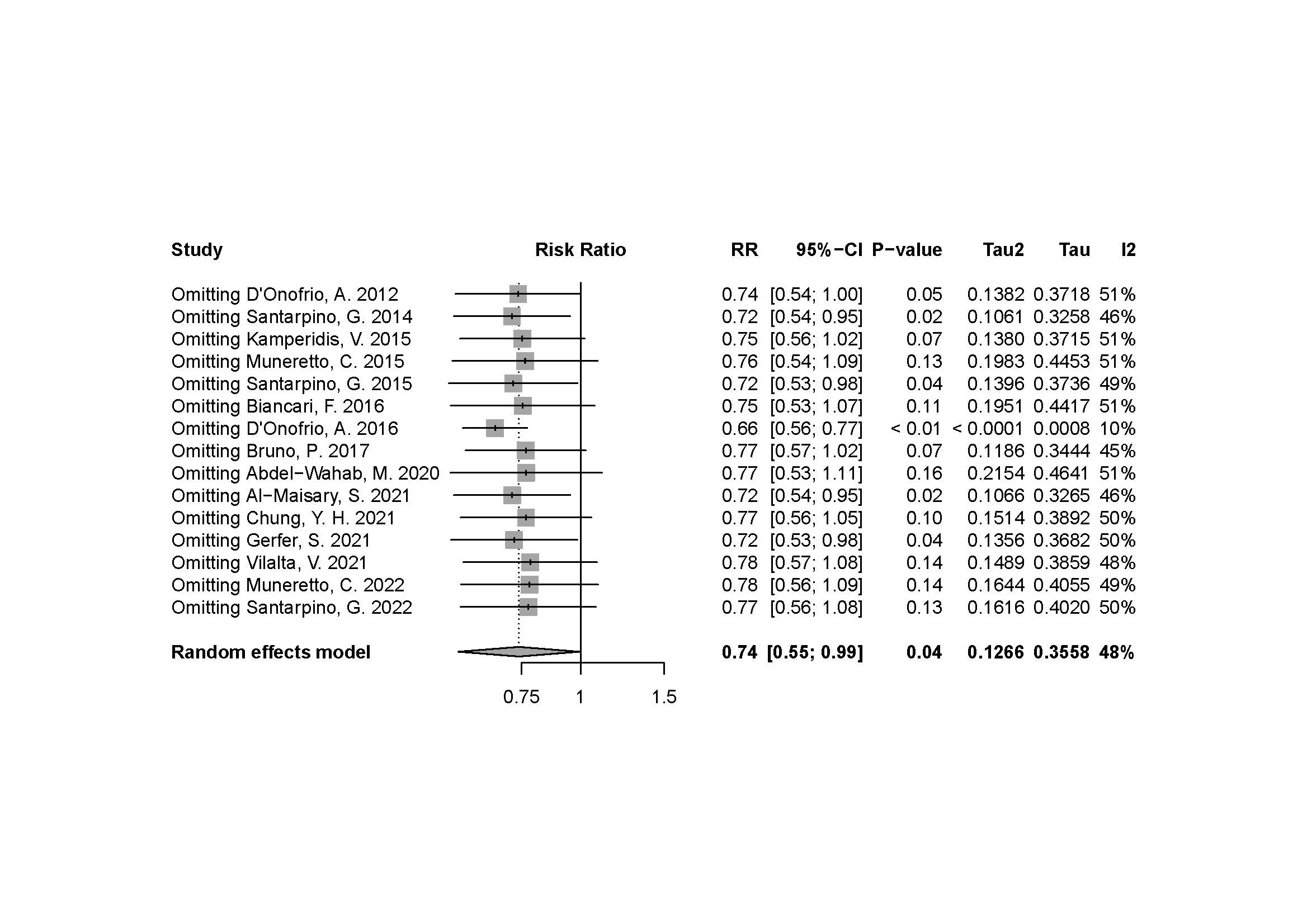


Supplementary Fig. 6 Sensitivity analyses for new PPI


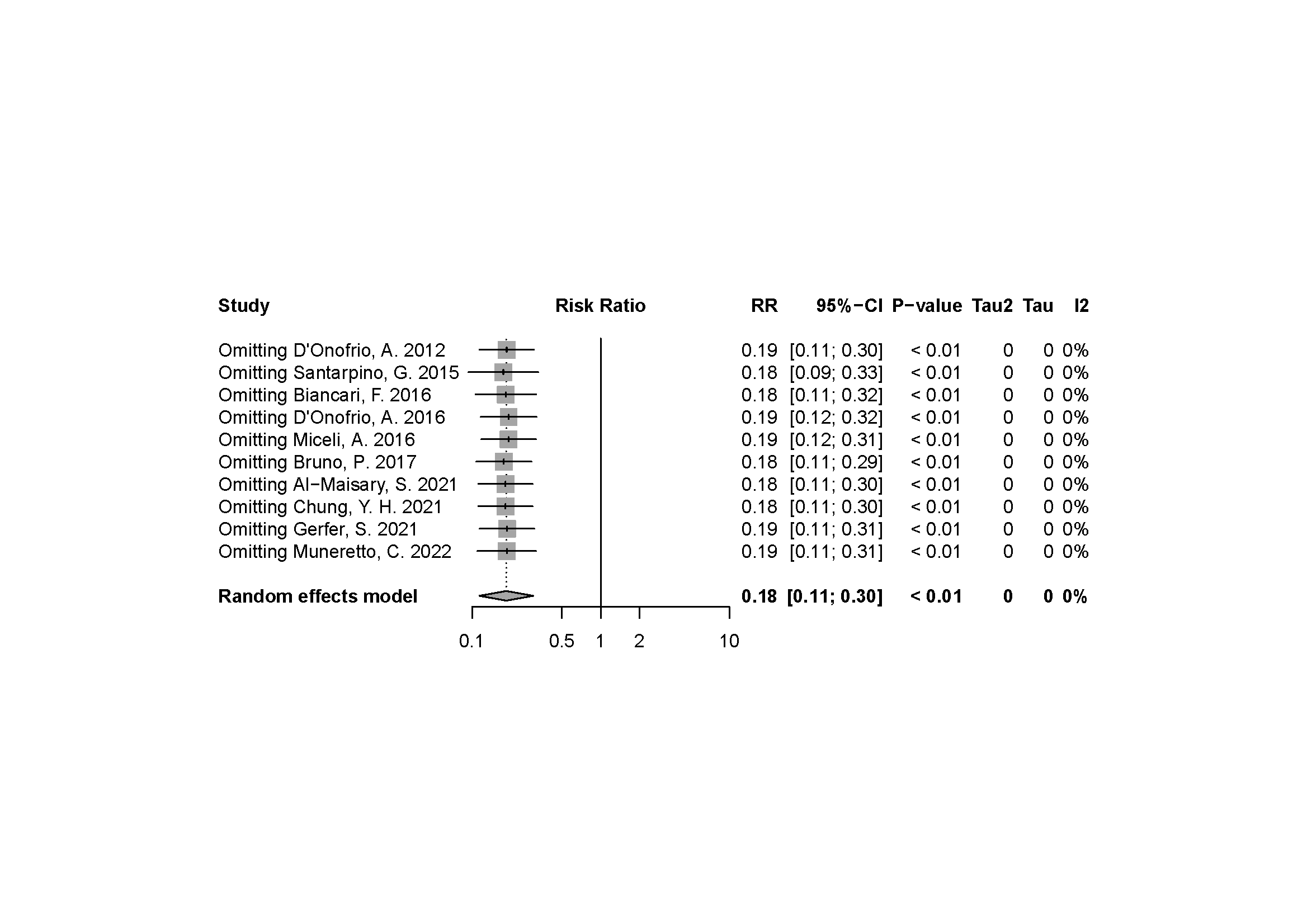


Supplementary Fig. 7 Sensitivity analyses for moderate-to-severe PVL


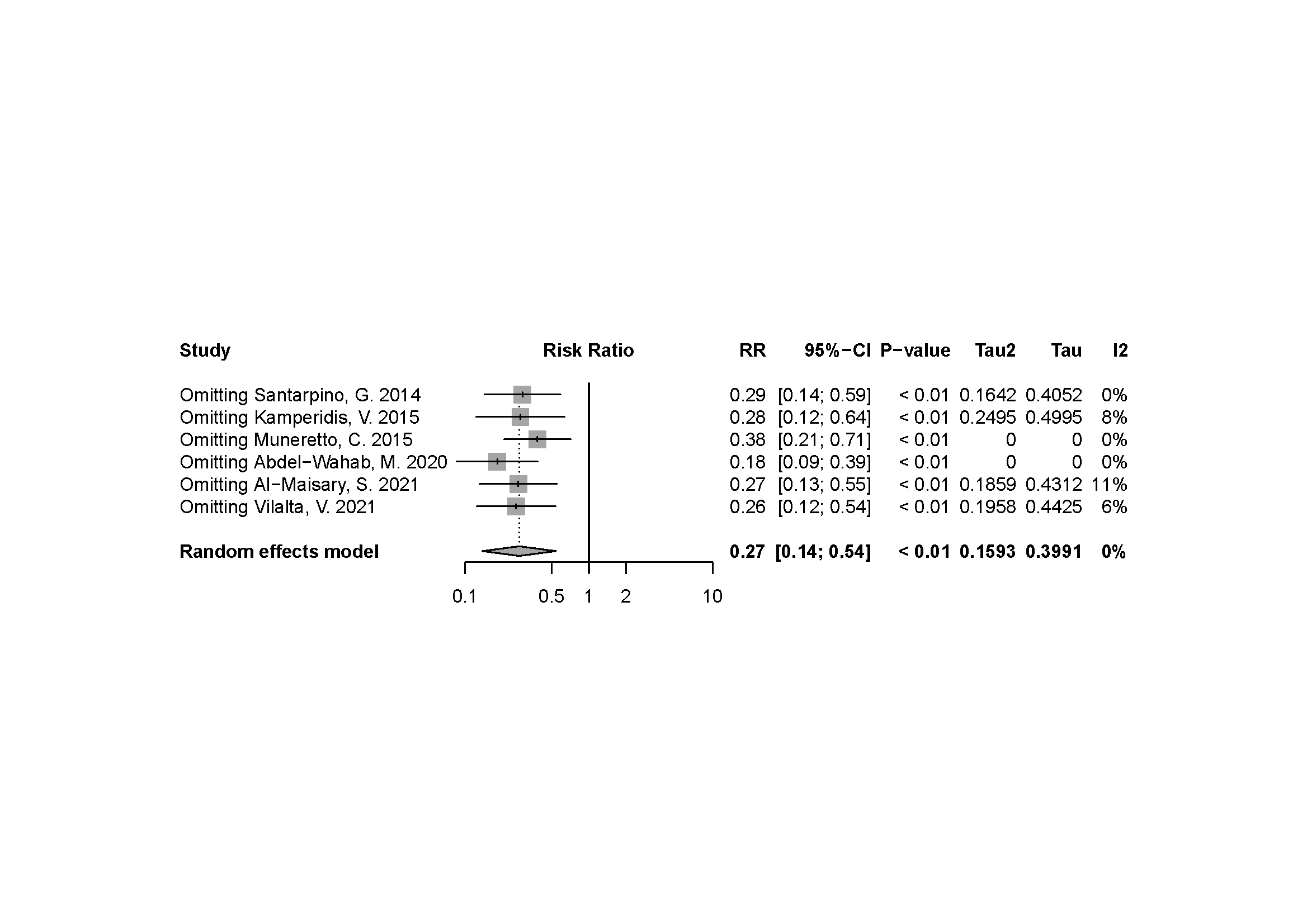


Supplementary Fig. 8 Sensitivity analyses for more-than-mild residual AR


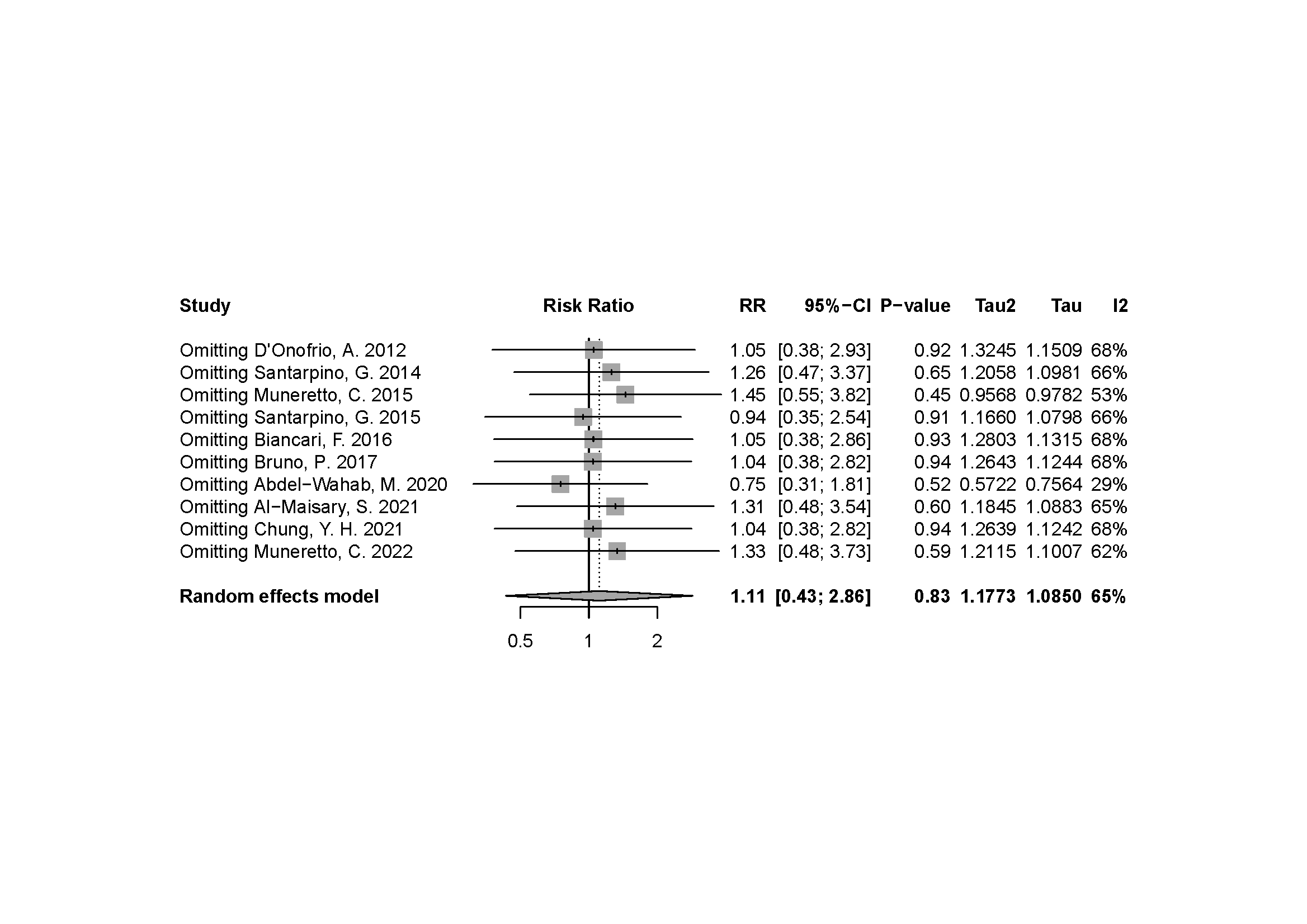


Supplementary Fig. 9 Sensitivity analyses for new renal replacement therapy


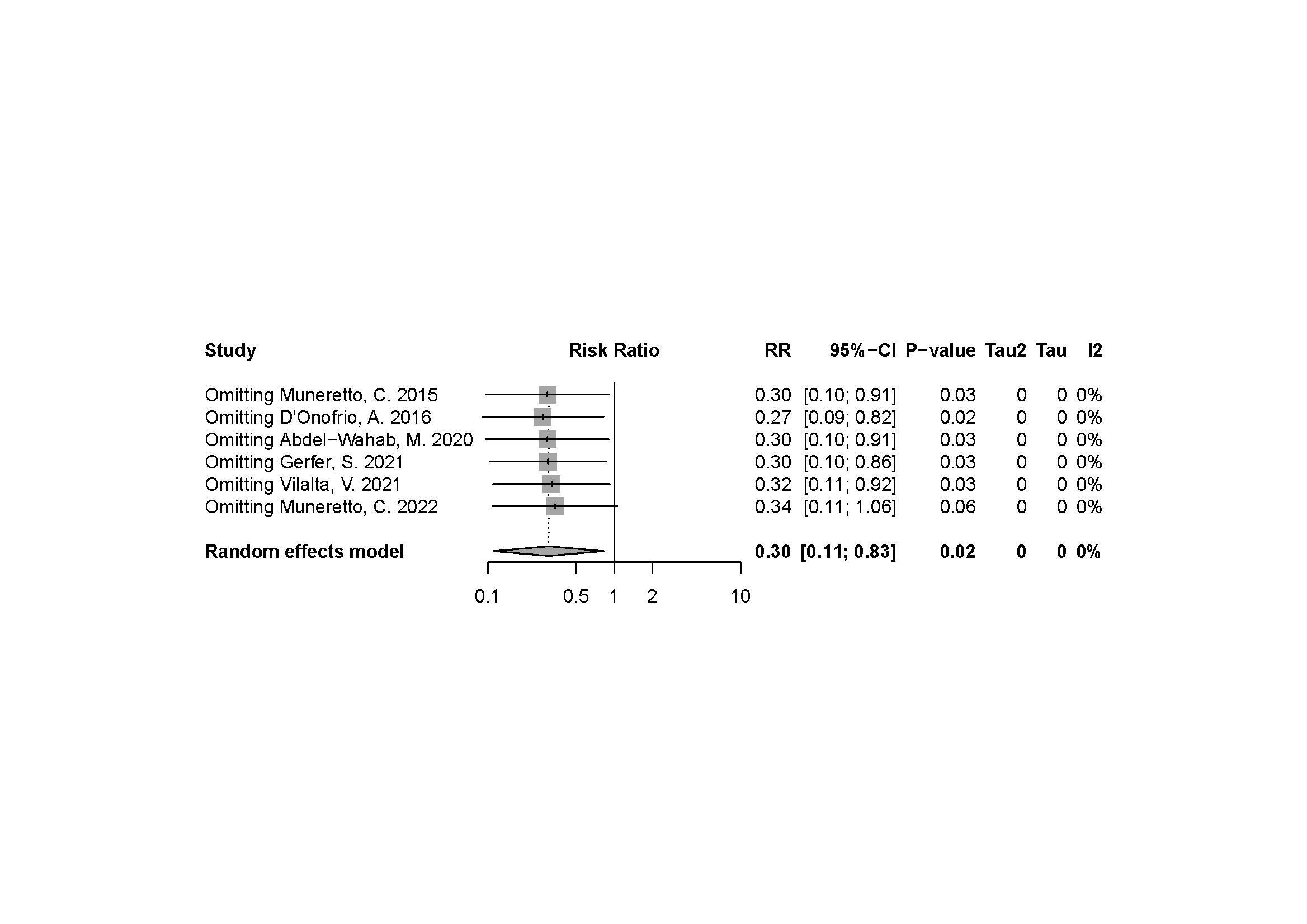


Supplementary Fig. 10 Sensitivity analyses for MI


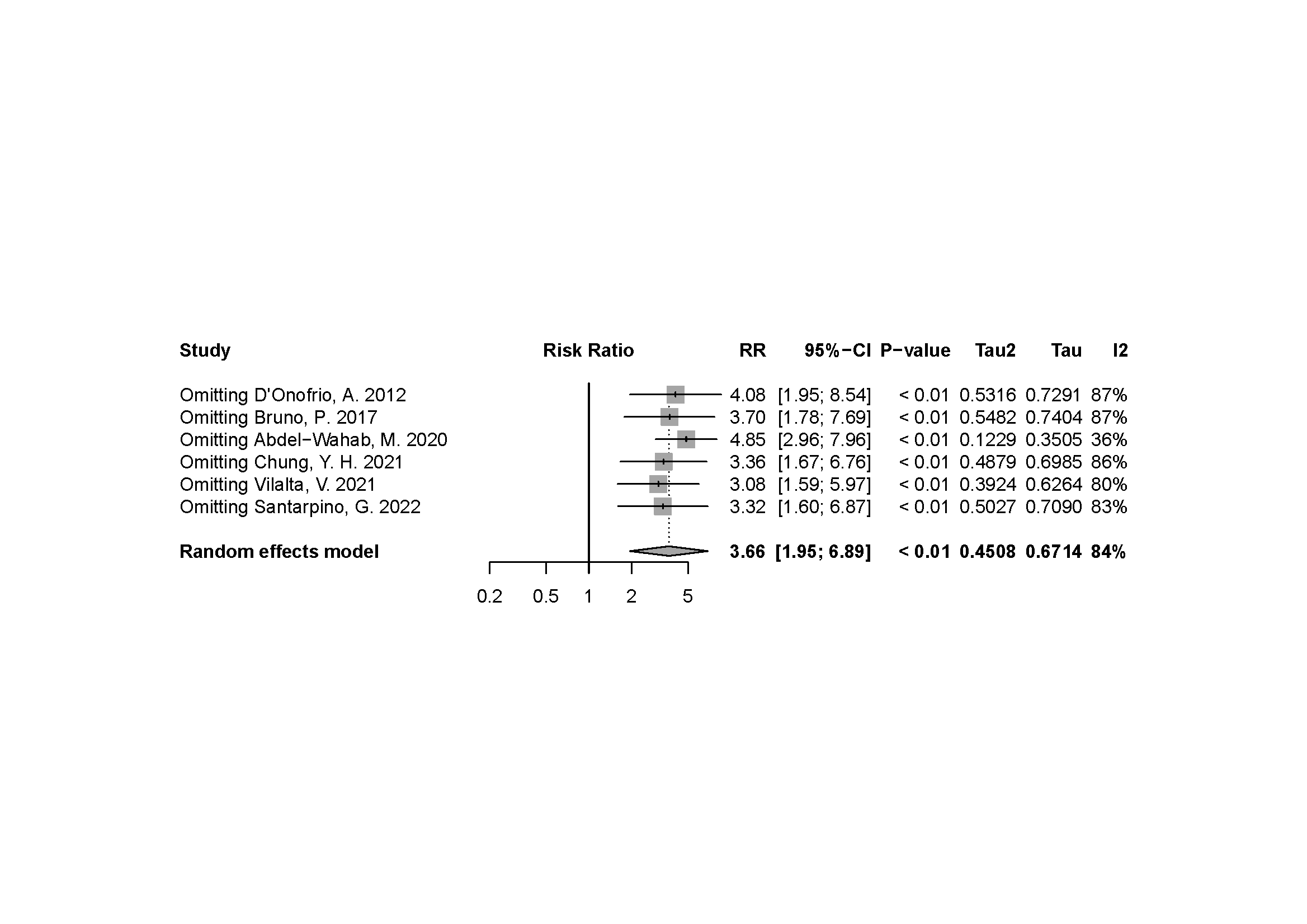


Supplementary Fig. 11 Sensitivity analyses for new AF


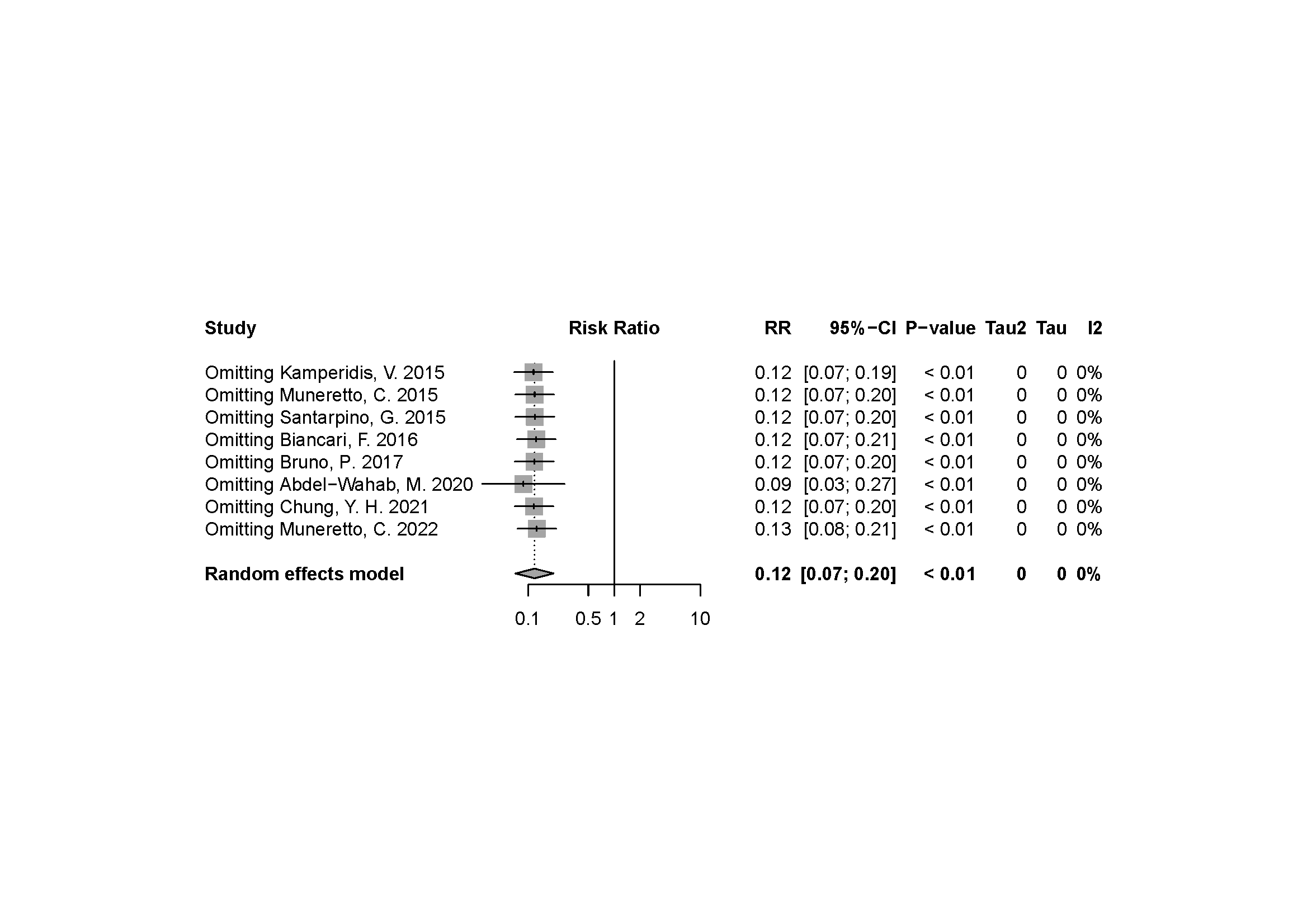


Supplementary Fig. 12 Sensitivity analyses for major vascular complication


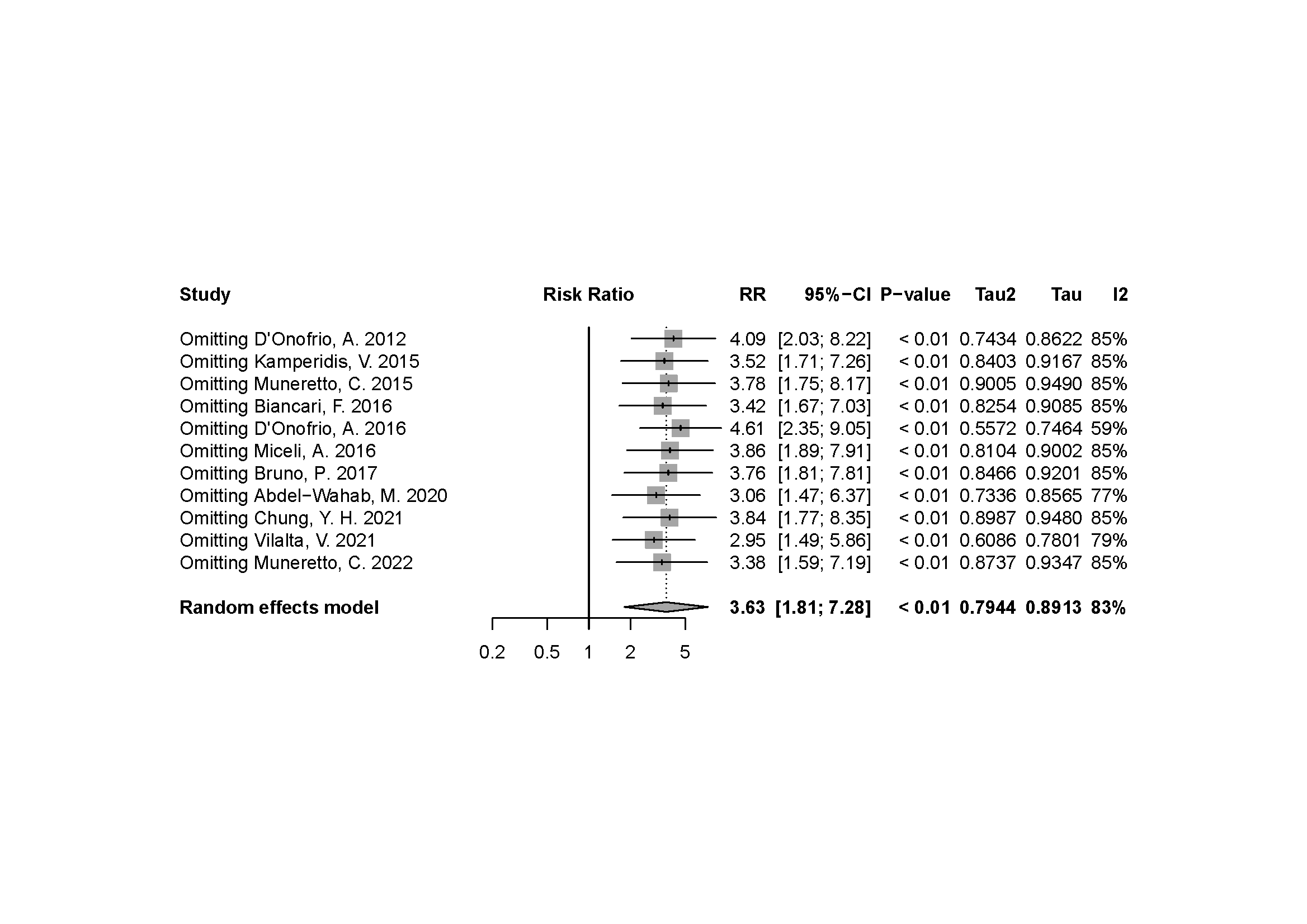


Supplementary Fig. 13 Sensitivity analyses for major or life-threatening bleeding event


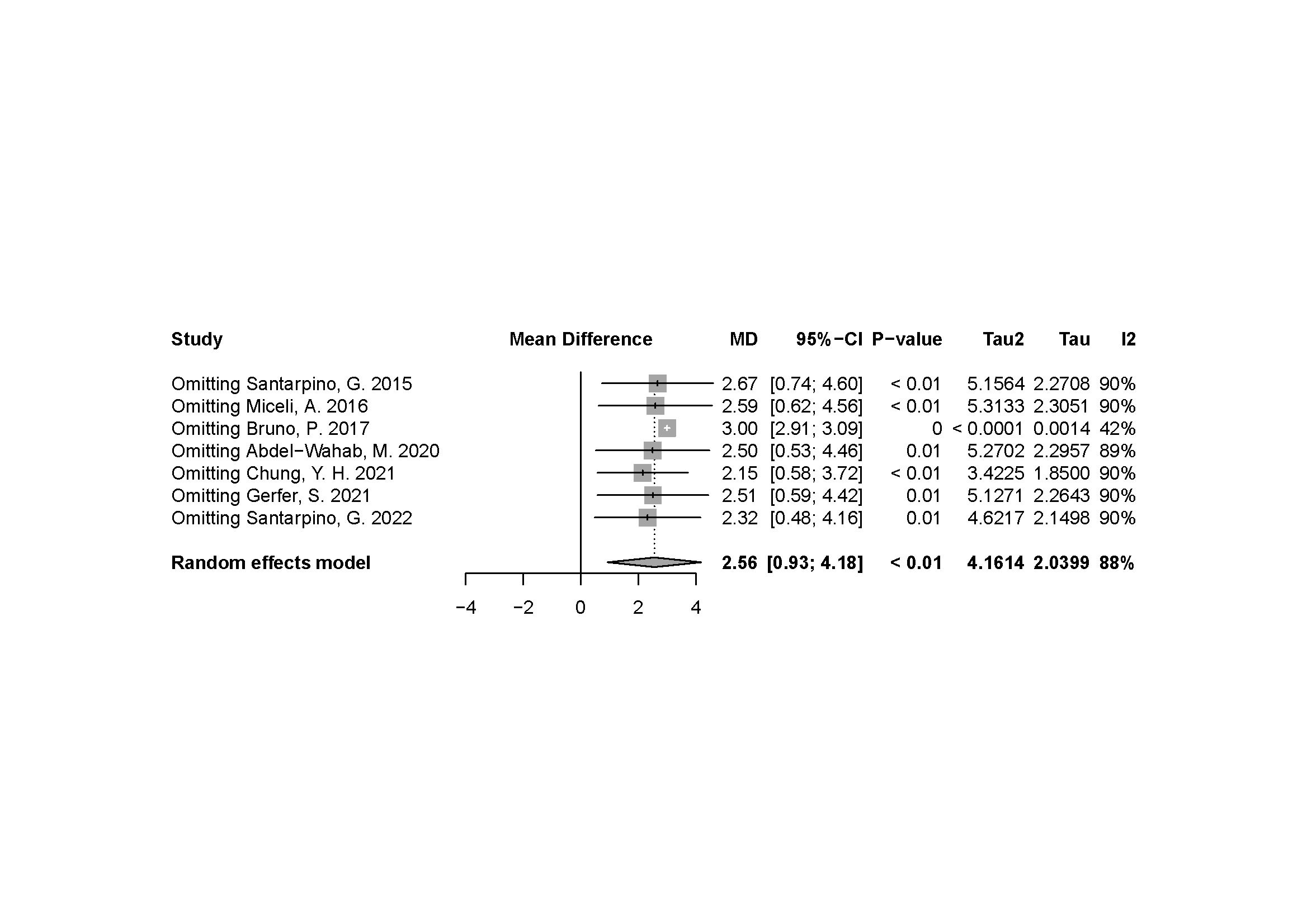


Supplementary Fig. 14 Sensitivity analyses for length of stay


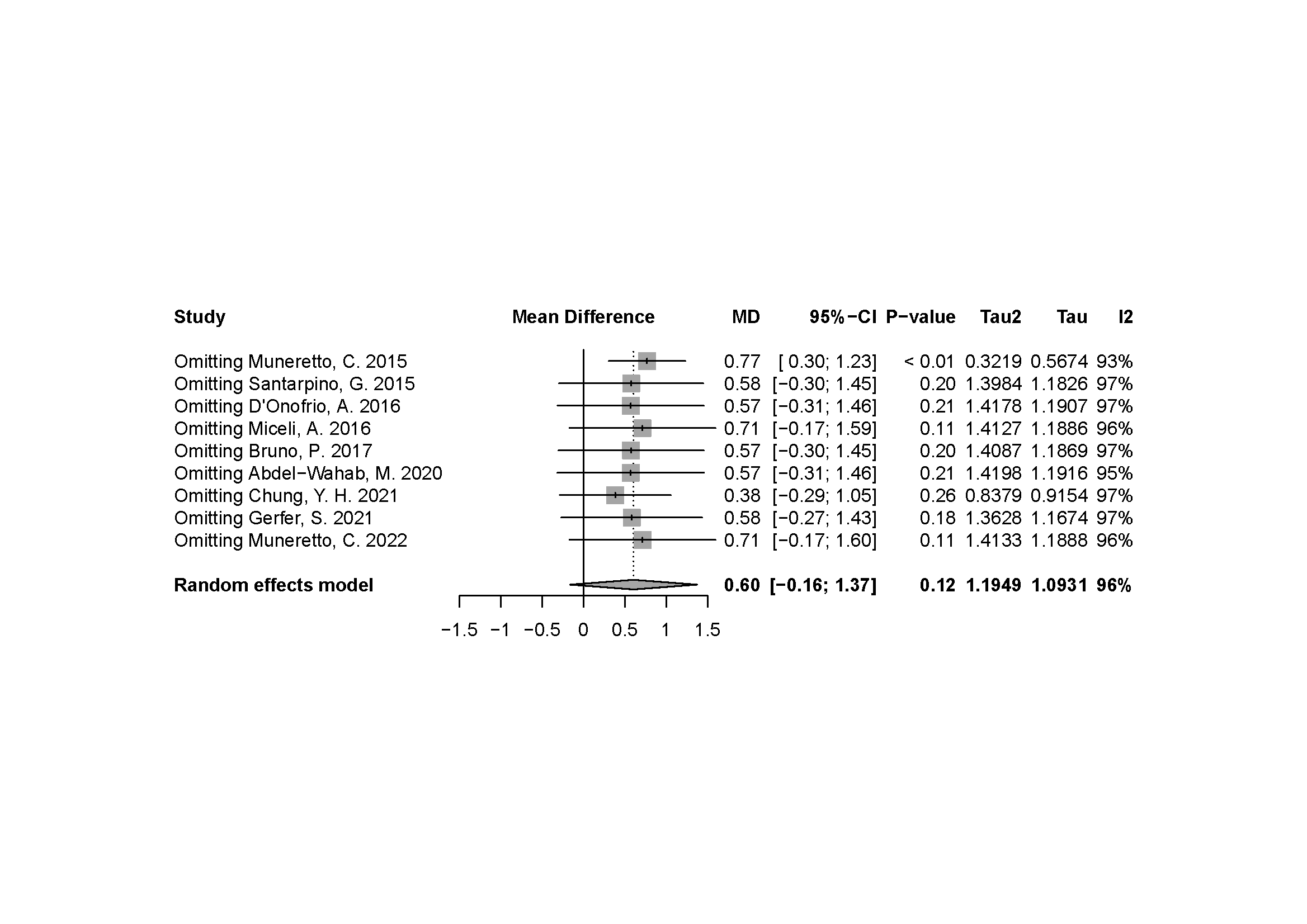


Supplementary Fig. 15 Sensitivity analyses for ICU length of stay


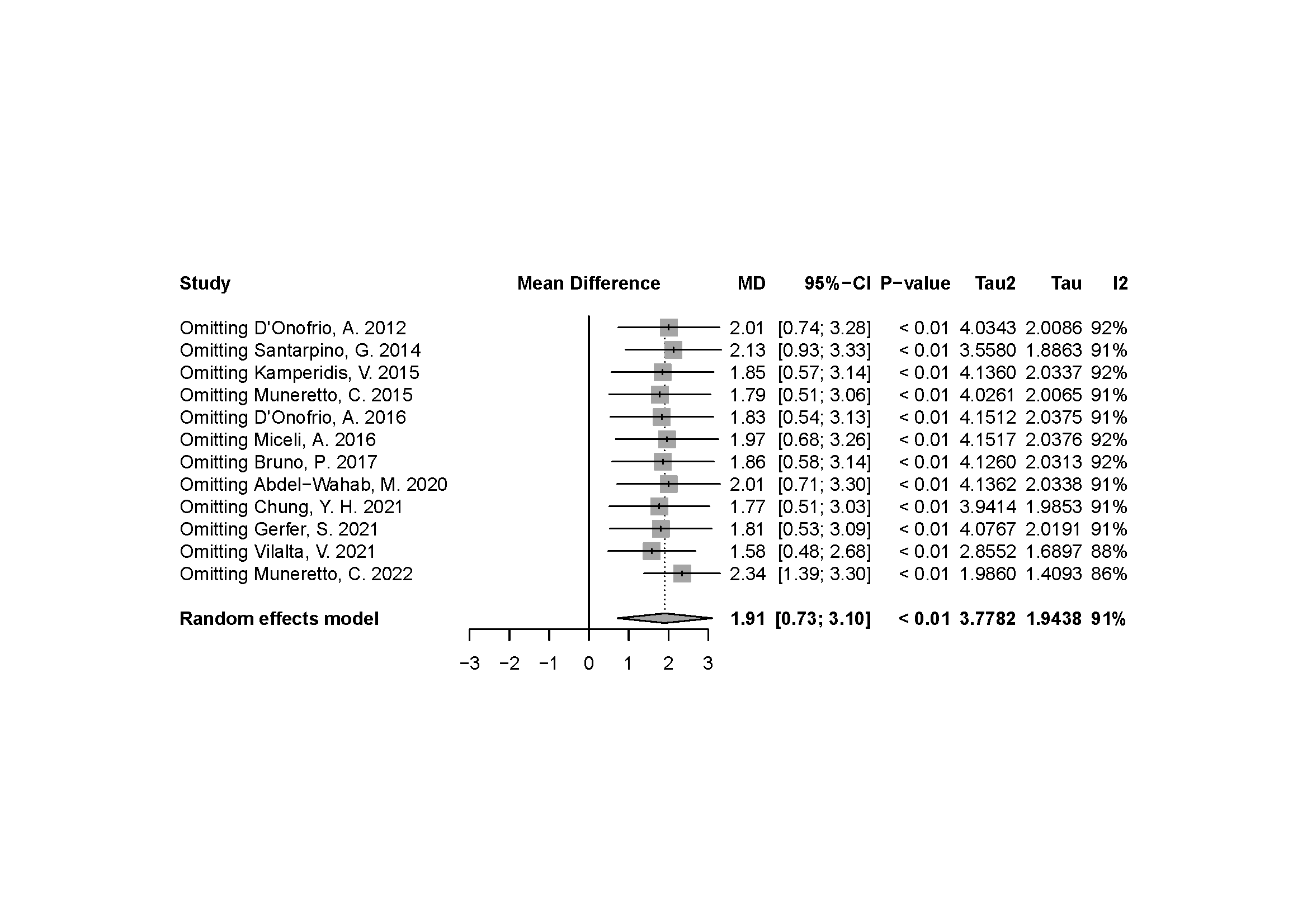


Supplementary Fig. 16 Sensitivity analyses for postoperative mean aortic gradient


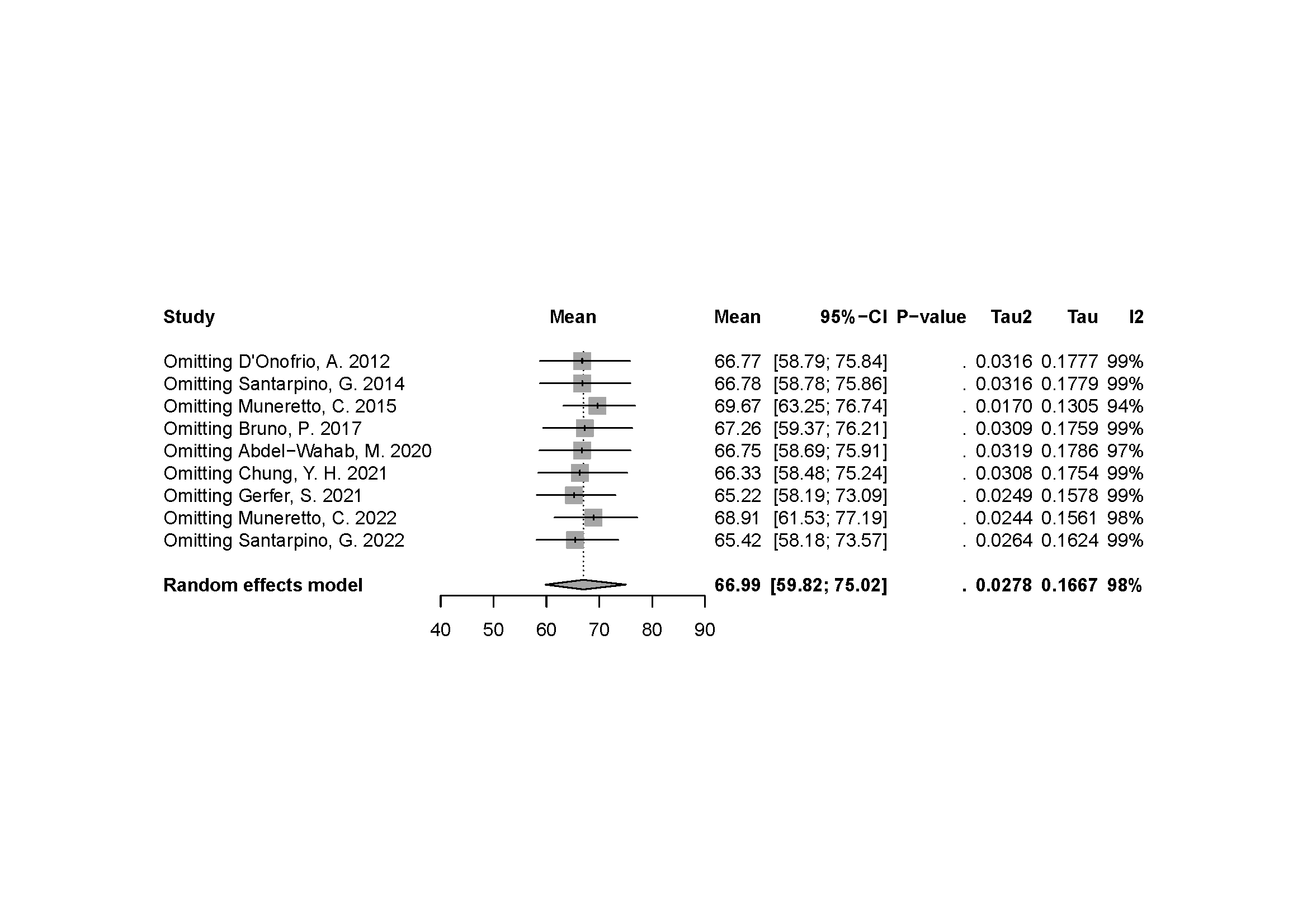


Supplementary Fig. 17 Sensitivity analyses for CPB time


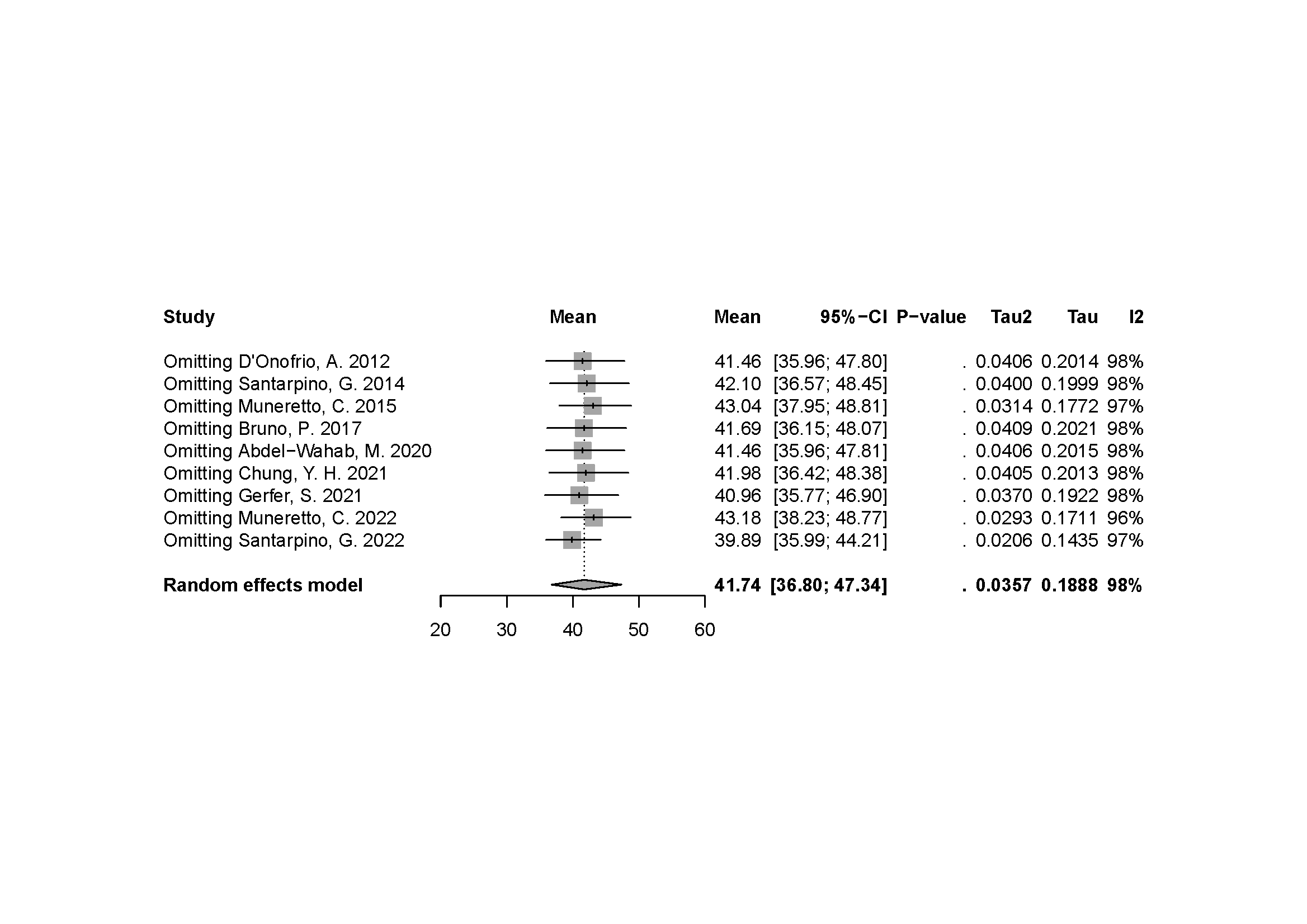


Supplementary Fig. 18 Sensitivity analyses for cross-clamp time
