# Supplementary material for: Comparison of Sutureless Aortic Valve Replacement and Transcatheter Aortic Valve Implantation: A Systematic Review and Meta-Analysis of Propensity Score Matching
Source: Rev Cardiovasc Med. 2024 Nov 4;25(11):391. doi: 10.31083/j.rcm2511391 (PMC11607518; doi:10.31083/j.rcm2511391)
Supplement: Supplementary file 1 [file 2153-8174-25-11-391-s1.zip › Supplementary 2-funnel plot analysis.docx]

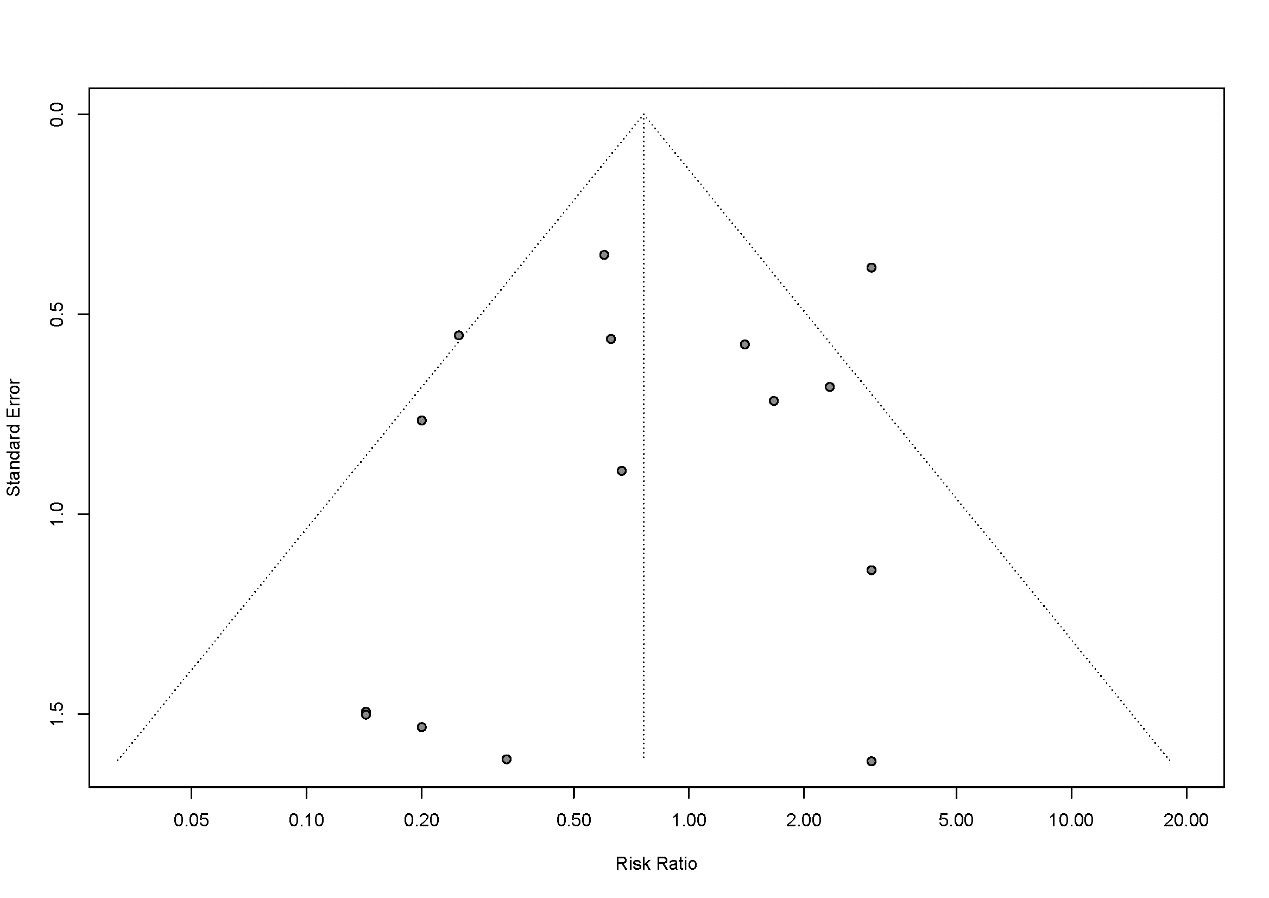


Supplementary Fig. 1 Funnel plot analysis for 30-day mortality


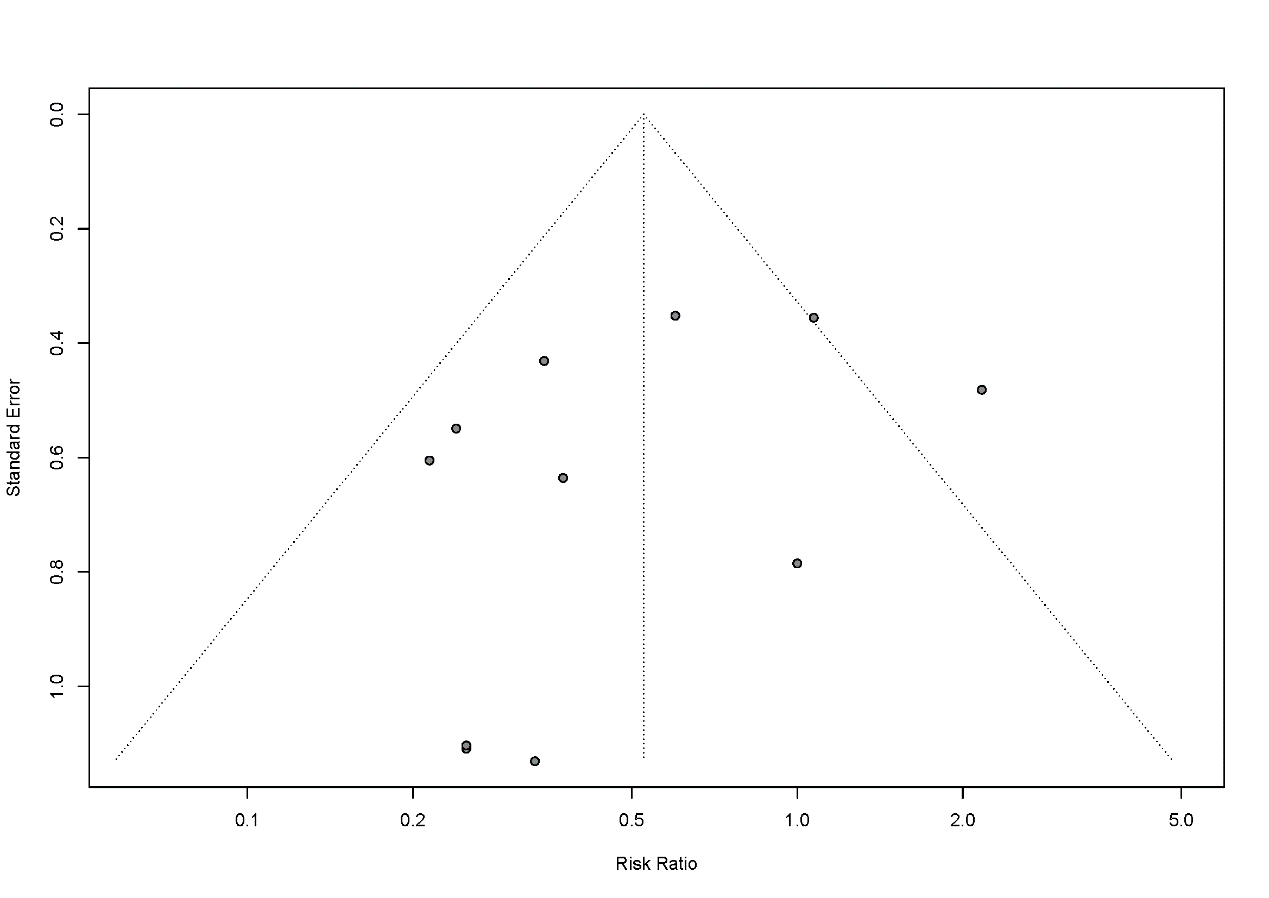


Supplementary Fig. 2 Funnel plot analysis for 1-year mortality


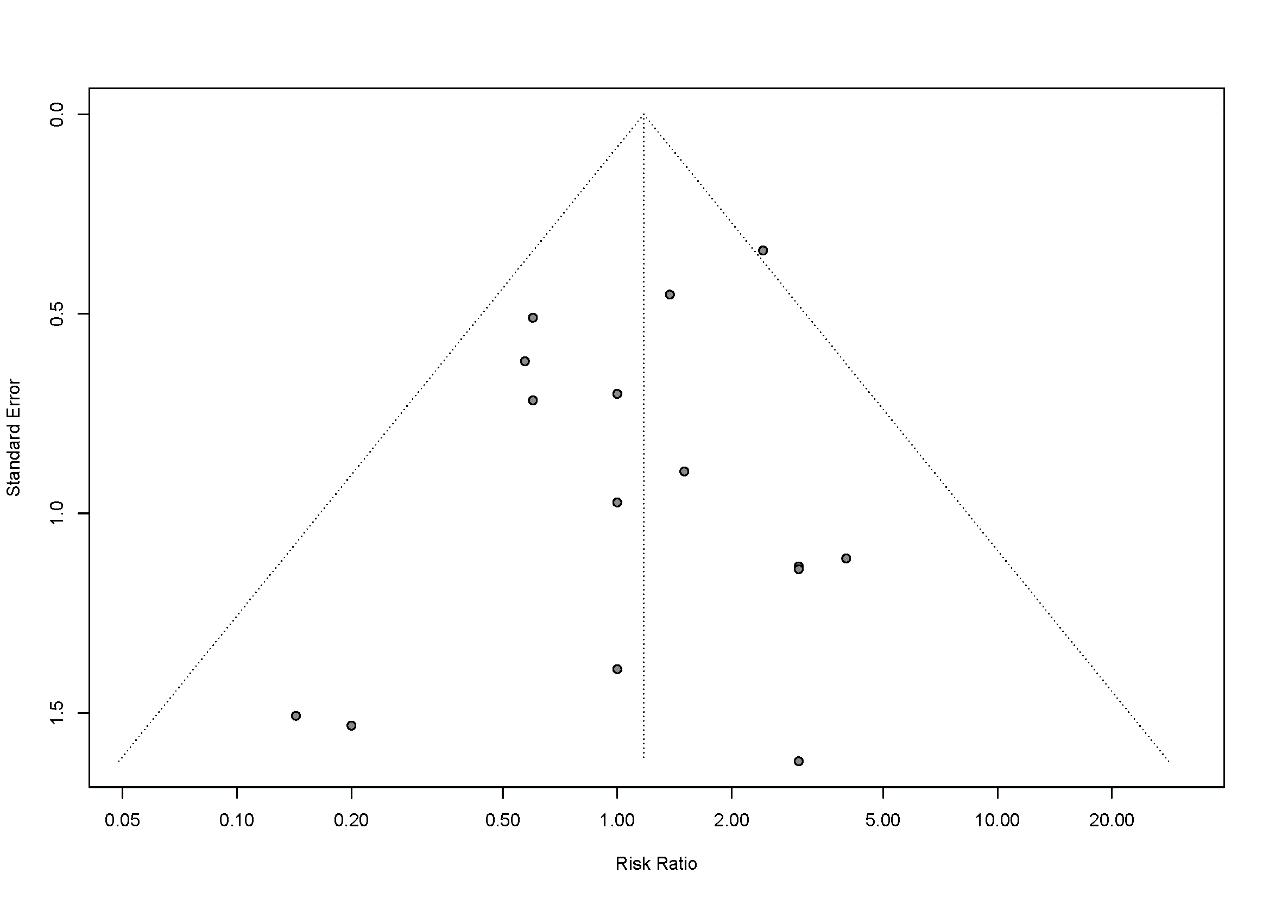


Supplementary Fig. 3 Funnel plot analysis for stroke


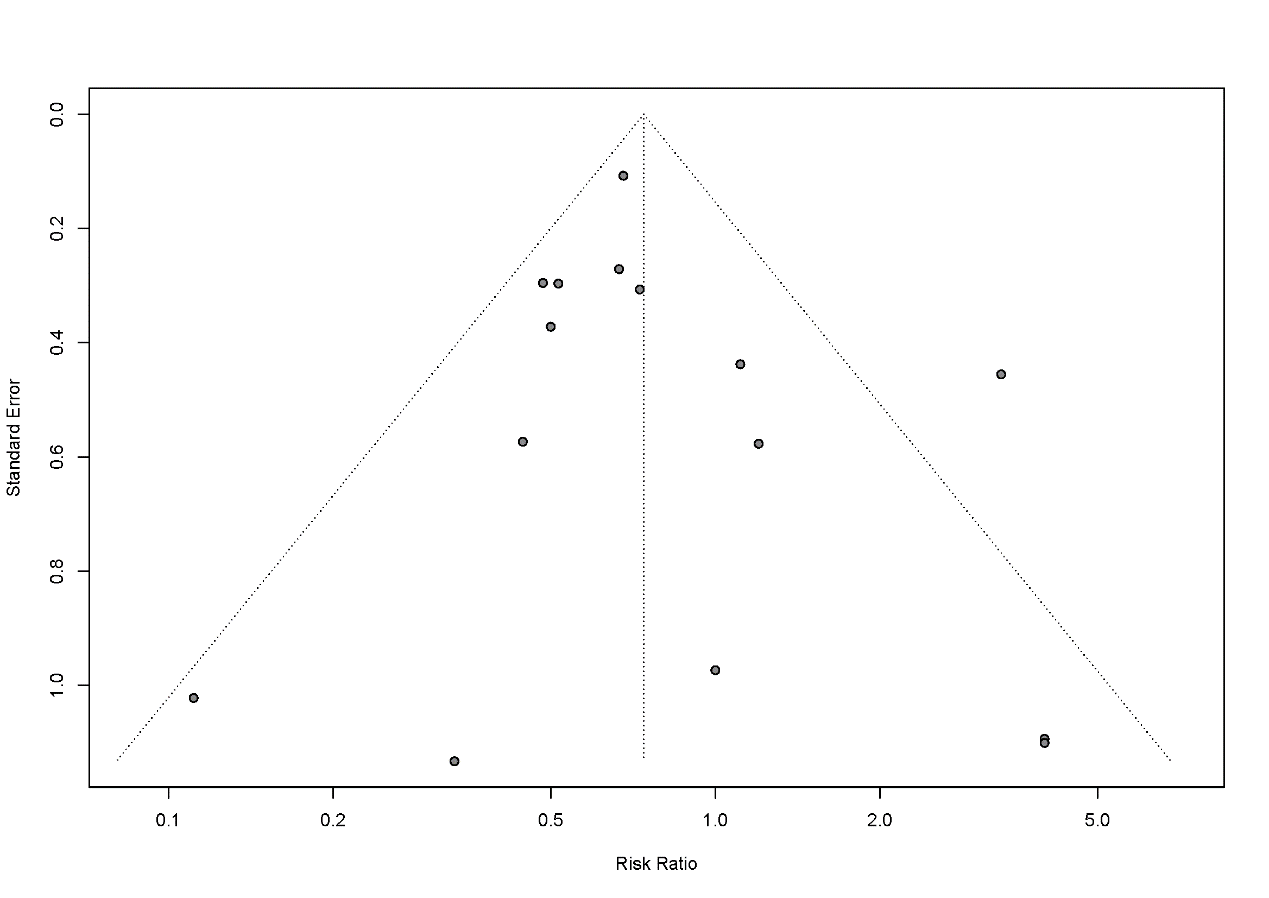


Supplementary Fig. 4 Funnel plot analysis for new PPI


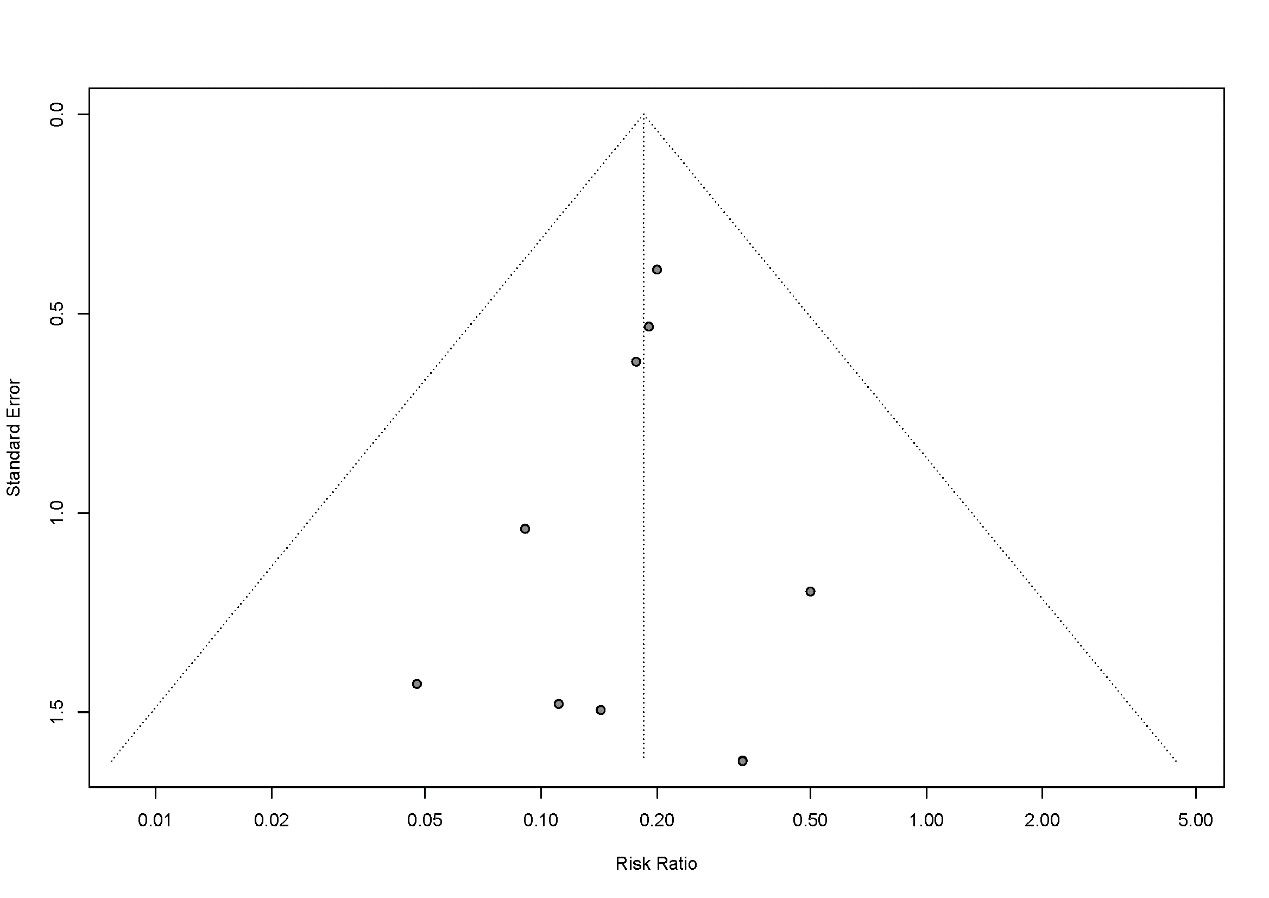


Supplementary Fig. 5 Funnel plot analysis for moderate-to-severe PVL


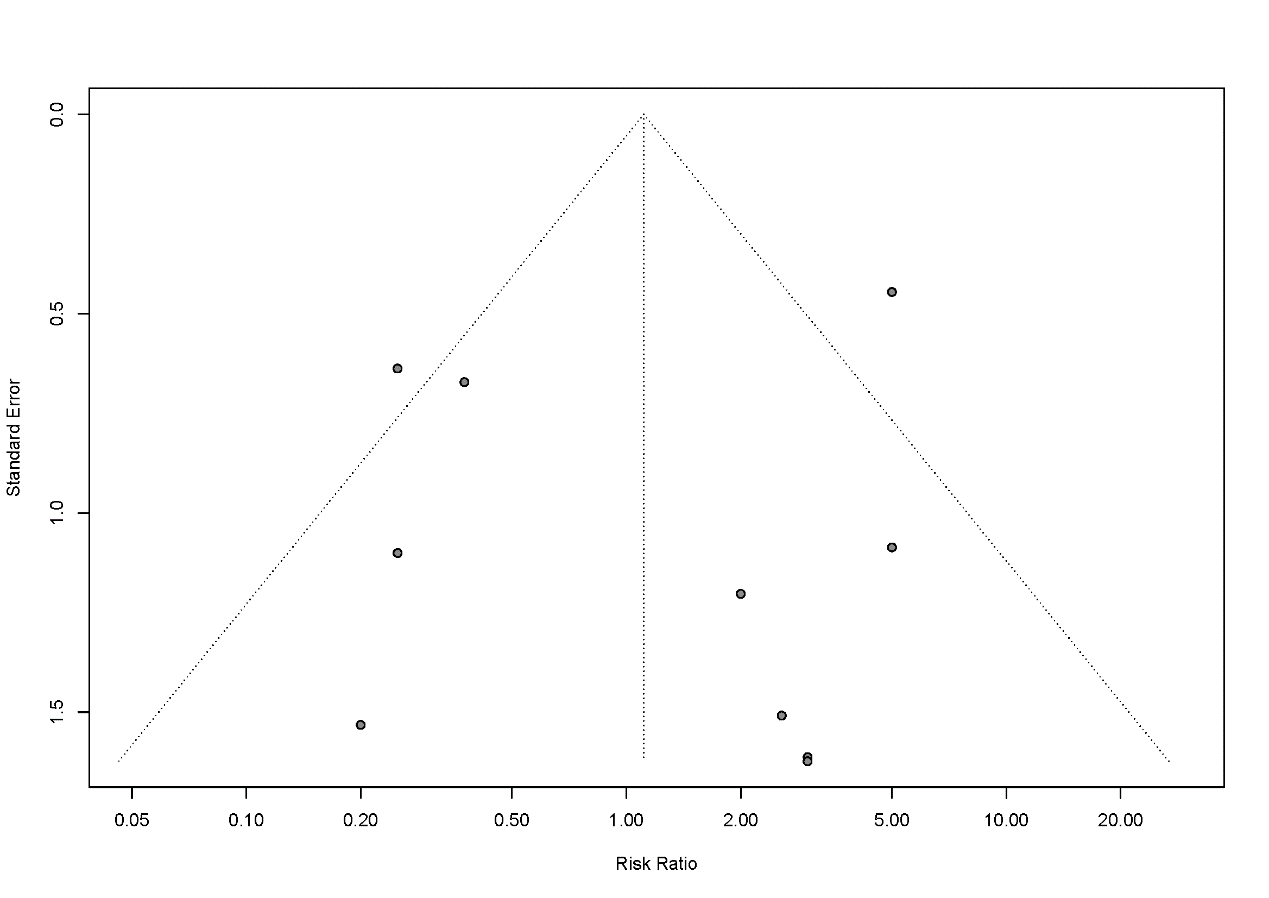


Supplementary Fig. 6 Funnel plot analysis for new renal replacement therapy


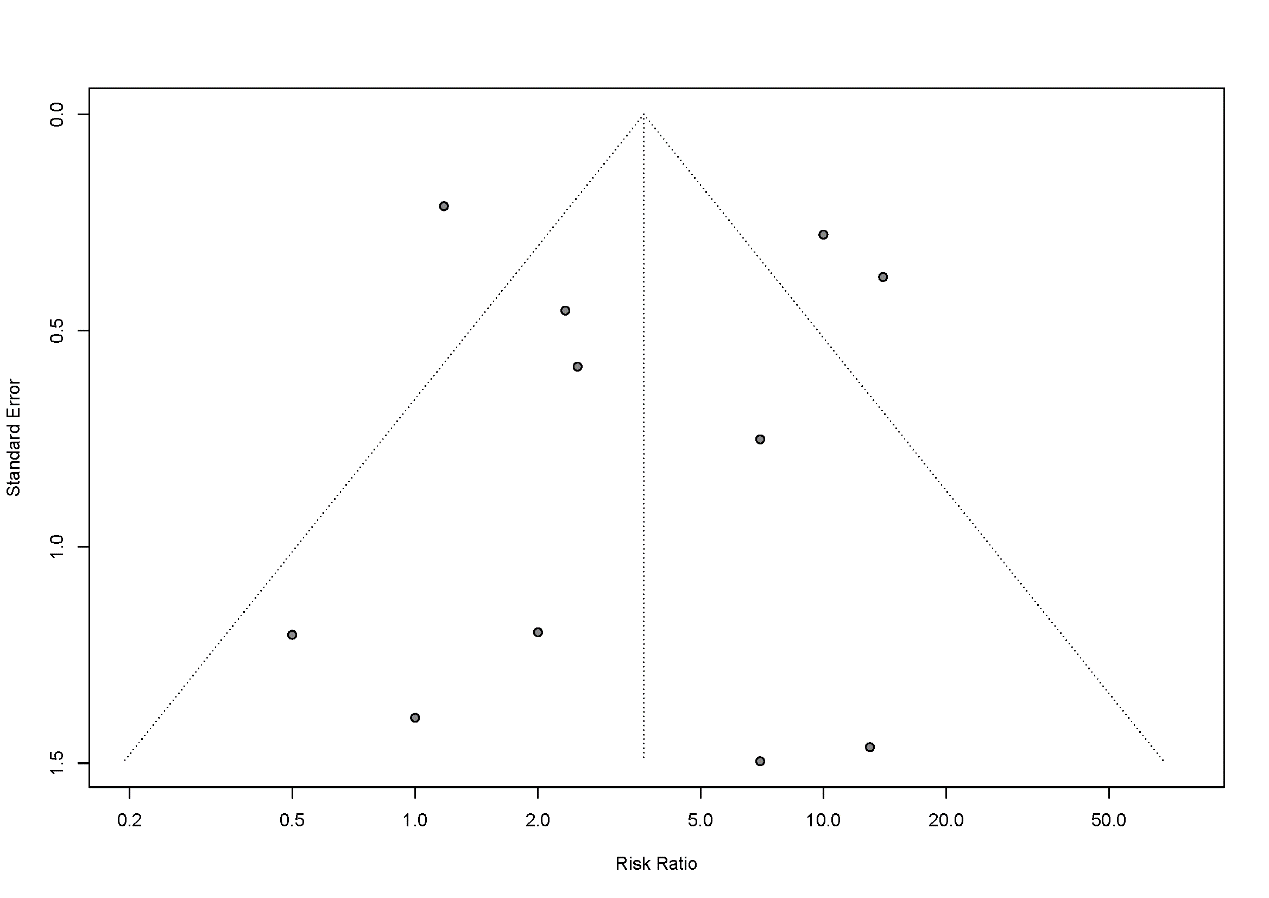


Supplementary Fig. 7 Funnel plot analysis for major or life-threatening bleeding event


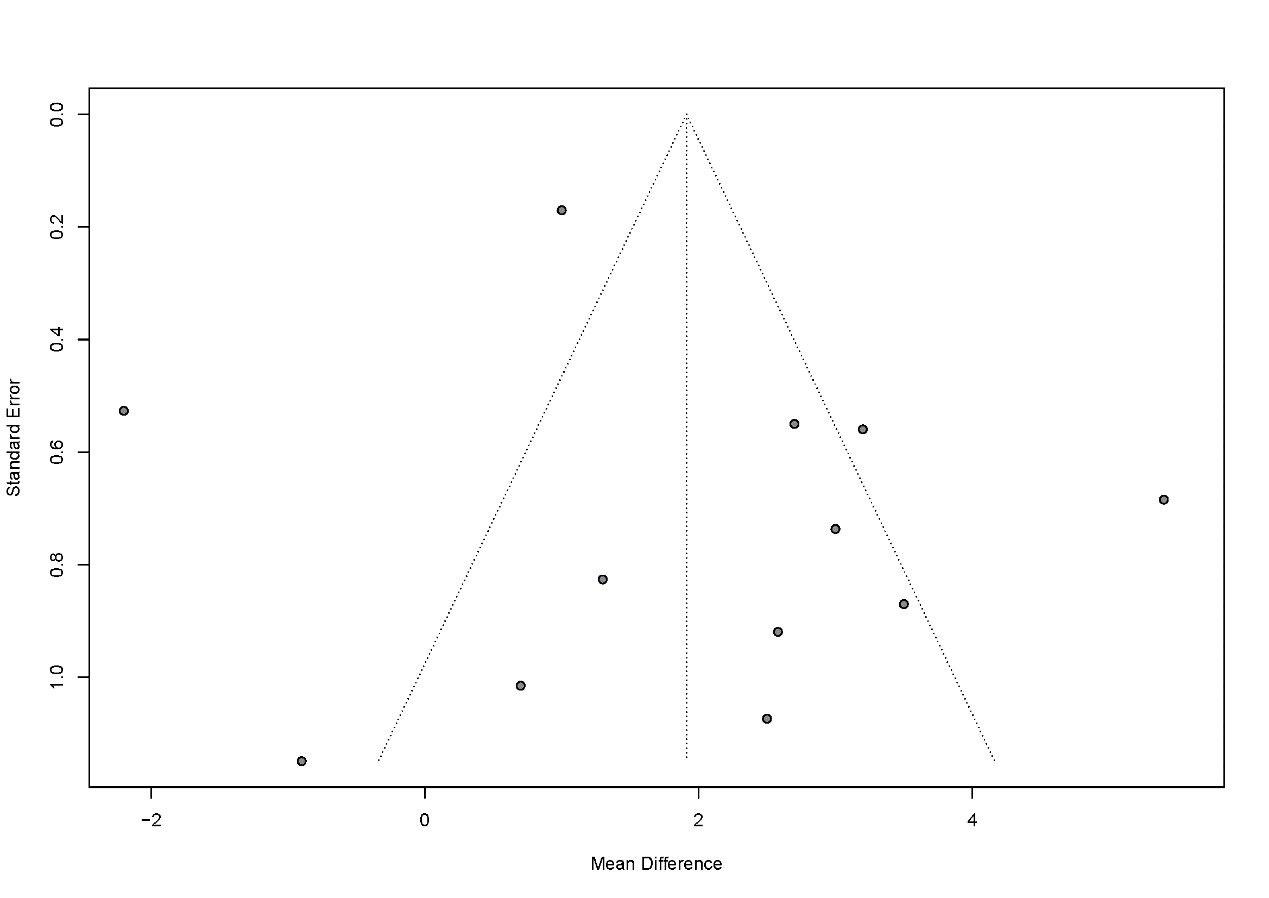


Supplementary Fig. 8 Funnel plot analysis for postoperative mean aortic gradient
